# Molecular glues for manipulating enzymes: trypsin inhibition by benzamidine-conjugated molecular glues†
†Electronic supplementary information (ESI) available: Synthesis of TEG–BA, Glue_*n*_–BA, ^*m*^Glue_*n*_–BA and Glue_*n*_–Ph; ^1^H NMR, ^13^C NMR, MALDI-TOF MS, electronic absorption, and CD spectra; zeta potential distributions; SLS plots; DLS histograms; and related experimental procedures. See DOI: 10.1039/c5sc00524h



**DOI:** 10.1039/c5sc00524h

**Published:** 2015-03-18

**Authors:** Rina Mogaki, Kou Okuro, Takuzo Aida

**Affiliations:** a Department of Chemistry and Biotechnology , School of Engineering , The University of Tokyo , 7-3-1 Hongo, Bunkyo-ku , Tokyo 113-8656 , Japan . Email: okuro@macro.t.u-tokyo.ac.jp ; Email: aida@macro.t.u-tokyo.ac.jp ; Tel: +81-3-5841-7251; b RIKEN Center for Emergent Matter Science , 2-1 Hirosawa , Wako , Saitama 351-0198 , Japan

## Abstract

Water-soluble bioadhesive polymers bearing multiple guanidinium ion (Gu^+^) pendants at their side-chain termini (Glue_*n*_–BA, *n* = 10 and 29) that were conjugated with benzamidine (BA) as a trypsin inhibitor were developed. The Glue_*n*_–BA molecules are supposed to adhere to oxyanionic regions of the trypsin surface, even in buffer, *via* a multivalent Gu^+^/oxyanion salt-bridge interaction, such that their BA group properly blocks the substrate-binding site. In fact, Glue_10_–BA and Glue_29_–BA exhibited 35- and 200-fold higher affinities for trypsin, respectively, than a BA derivative without the glue moiety (TEG–BA). Most importantly, Glue_10_–BA inhibited the protease activity of trypsin 13-fold more than TEG–BA. In sharp contrast, ^*m*^Glue_27_–BA, which bears 27 Gu^+^ units along the main chain and has a 5-fold higher affinity than TEG–BA for trypsin, was inferior even to TEG–BA for trypsin inhibition.

## Abstract 2

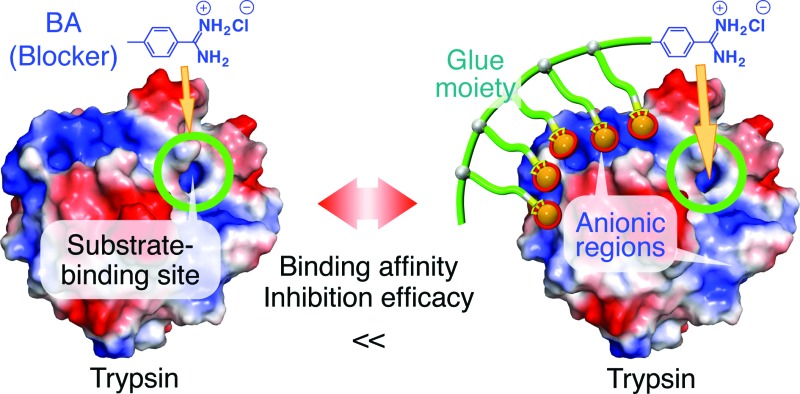
The inhibitory effect of benzamidine as blocker on the protease activity of trypsin is enhanced by covalent conjugation with bioadhesive molecular glue.

## Introduction

**Fig. 1:**
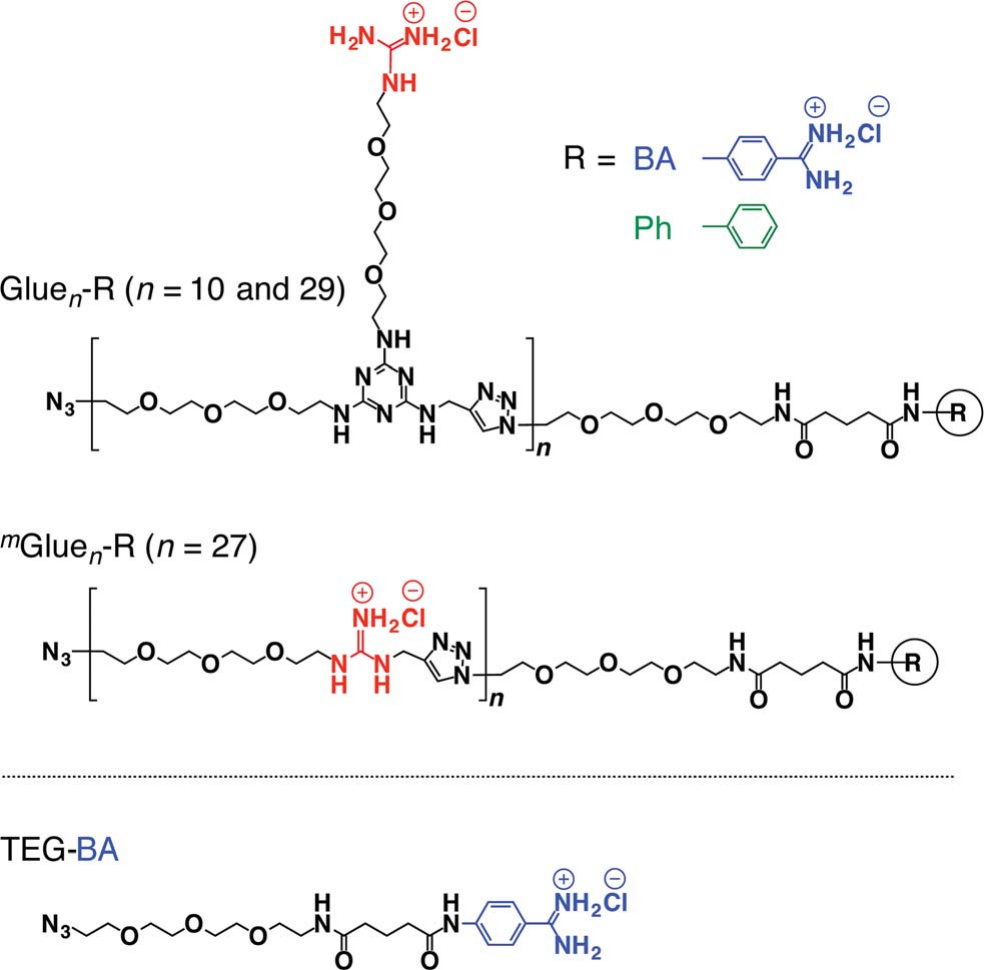
Schematic structures of bioadhesive molecular glues Glue_*n*_–R (*n* = 10 and 29) and ^*m*^Glue_*n*_–R (*n* = 27) conjugated with benzamidine (R = BA) as a trypsin inhibitor, and those of the reference molecular glue Glue_*n*_–Ph without an inhibitory terminus and TEGylated benzamidine (TEG–BA) without the glue moiety.

**Fig. 2:**
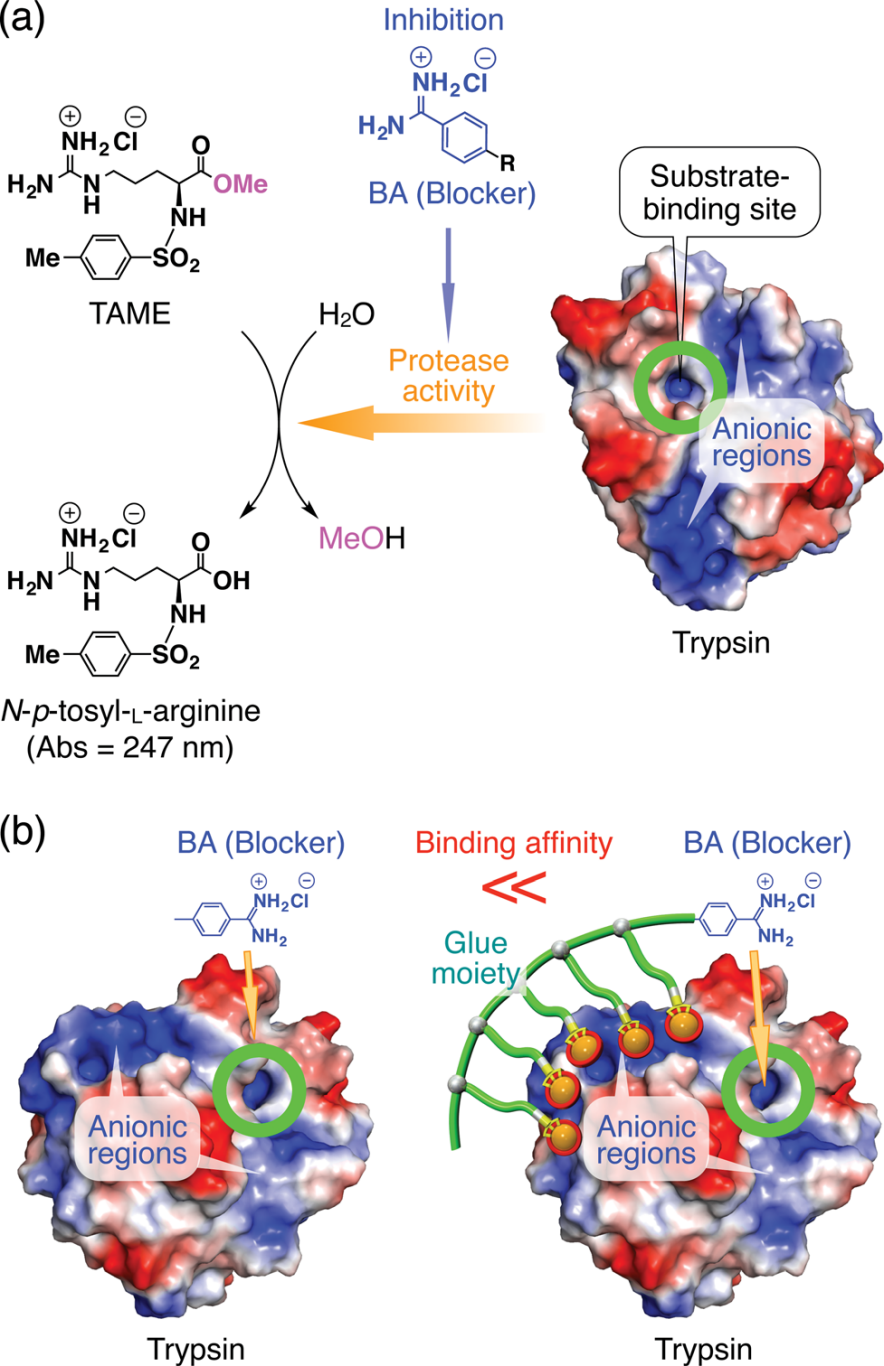
(a) Schematic illustration of the trypsin-catalyzed hydrolysis of *N-p*-tosyl-l-arginine methyl ester (TAME) to *N-p*-tosyl-l-arginine, exhibiting its characteristic absorption at 247 nm.^10,11^ Benzamidine (BA) derivatives are known to bind to the substrate-binding site of trypsin and inhibit its enzymatic activity.^10^ Trypsin has oxyanionic regions on its surface (blue-coloured) in proximity to the substrate-binding site, which allow the glue moiety of Glue_*n*_–BA to adhere. (b) Schematic representation of how the inhibitory effect of the blocking unit BA on the protease activity of trypsin is enhanced by conjugation with molecular glue.

If the behaviours of enzymes are manipulated by noncovalent interactions,^1–4^ one may possibly alter their functions and eventually control related biological events. In this context, one ambitious goal might be to noncovalently operate enzymes such that they perform different functions from their original tasks. In early studies, for instance, amphiphilic molecules have been utilized to introduce enzymes to non-aqueous media in order to expand the range of substrates.^5^ Nevertheless, from a pharmacological viewpoint, noncovalent enhancement or attenuation of certain enzymatic activities^6–9^ is a highly important and challenging subject. As a proof-of-concept study, we developed a benzamidine (BA) derivative appended with a particular bioadhesive polymer, *i.e.*, molecular glue (Glue_*n*_–BA, Fig. 1), which bears at its side-chain termini multiple guanidinium ion (Gu^+^) pendants that can be salt-bridged with oxyanionic groups on target protein surfaces. BA is known to inhibit the protease activity of trypsin by blocking its substrate-binding site (Fig. 2a).^10^ In proximity to this binding site, trypsin has oxyanionic regions^11^ (blue-coloured) that allow the glue moiety of Glue_*n*_–BA to adhere (Fig. 2a). Hence, we envisioned that Glue_*n*_–BA could inhibit the protease activity of trypsin much more than a BA derivative without the glue moiety such as TEG–BA (Fig. 1), if the adhesion of the glue moiety (Glue_*n*_) does not hamper the appropriate BA positioning toward the active site (Fig. 2b).

We have developed a series of dendritic molecular glues that bear multiple guanidinium ion (Gu^+^) pendants in the periphery of their water-soluble dendritic scaffolds.^12–16^ Such dendritic molecular glues tightly adhere to proteins,^12–14^ phospholipid membranes^15^ and clay nanosheets^16^ in aqueous media *via* the formation of multiple salt bridges between their Gu^+^ pendants and oxyanionic groups located on those targets. Most interesting along the line of this study was the observation that the photomechanical motion of an azobenzene-cored molecular glue can be transmitted to a phospholipid vesicular membrane *via* salt-bridge interactions and can consequently modulate its transmembrane ion permeation.^15^ This finding motivated us to extend the scope of the present study more to bio-related applications, *i.e.*, noncovalent manipulation of enzymes. Recently, we confirmed that non-dendritic, linear polymers bearing side-chain Gu^+^ pendants^17^ are readily accessible alternatives to our prototype dendritic molecular glues. Hence, in the present study, we designed linear Glue_*n*_–BA with short (*n* = 10) and long (*n* = 29) glue moieties (Fig. 1). In addition to TEG–BA as a reference, we also prepared ^*m*^Glue_*n*_–BA (*n* = 27, Fig. 1) carrying 27 Gu^+^ units along the polymer main chain. As highlighted in this article, Glue_10_–BA inhibited the trypsin activity much more than TEG–BA (Fig. 1) without the glue moiety, whereas ^*m*^Glue_27_–BA (Fig. 1) was inferior to TEG–BA despite the fact that it carries 27 Gu^+^ units and has a higher affinity than TEG–BA for trypsin.

## Results and discussion

Glue_*n*_–BA was synthesized using a “click” reaction^18–23^ between TEG–BA and a three-armed monomer containing Gu^+^, azide and alkyne moieties. The reaction mixture was subjected to preparative size exclusion chromatography to allow fractionation of Glue_10_–BA and Glue_29_–BA. ^*m*^Glue_*n*_–BA (average *n* = 27, Fig. 1) was synthesized in a similar fashion by polymerizing a Gu^+^-containing linear monomer with terminal azide and alkyne groups. The average molecular weights of Glue_*n*_–BA and ^*m*^Glue_*n*_–BA were estimated by ^1^H NMR spectroscopy and static light scattering (SLS) analysis (Table S1†).

**Fig. 3:**
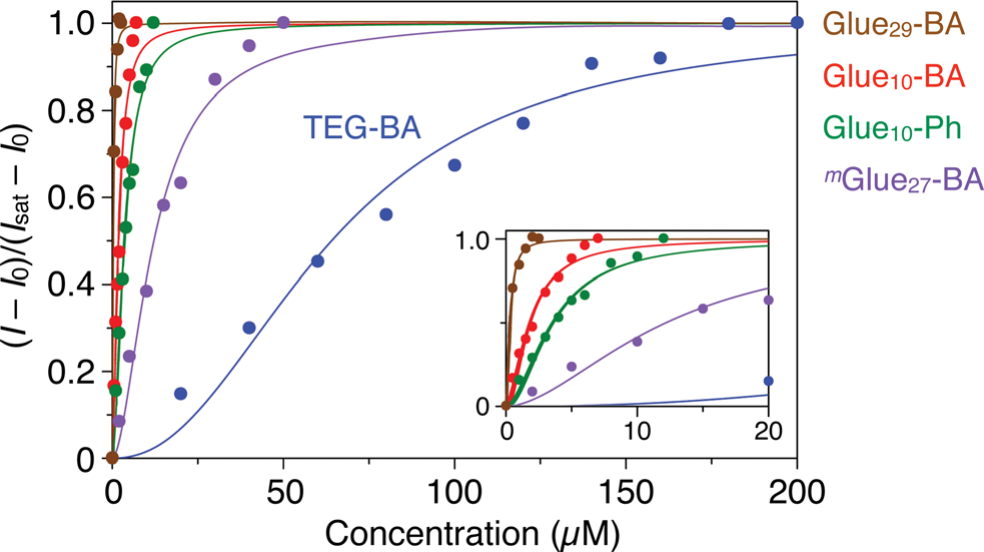
Circular dichroism (CD) spectral titration profiles of trypsin (5 μM) at 237 nm with molecular glues Glue_29_–BA (brown; 0–2.5 μM), Glue_10_–BA (red; 0–7 μM), Glue_10_–Ph (green; 0–12 μM) and ^*m*^Glue_27_–BA (purple; 0–50 μM), together with reference TEG–BA (blue; 0–200 μM), at 25 °C in Tris–HCl buffer (50 mM Tris–HCl, 10 mM CaCl_2_, pH 8.0). The fractions of bound trypsin were calculated from (*I* – *I*_0_)/(*I*_sat_ – *I*_0_), where *I*_0_, *I* and *I*_sat_ represent the CD intensities before titration, observed with titrants and at the saturation point, respectively.

We first investigated the effect of conjugation of molecular glues to BA on the binding affinity for trypsin. Trypsin is known to alter its conformation upon interaction with metal ions,^24^ polymers^25^ and proteins,^26^ resulting in circular dichroism (CD) spectral changes. Upon mixing with Glue_10_–BA, trypsin also changed its CD spectrum. As shown in Fig. S10c,† the CD intensity of trypsin (5 μM) at 237 nm decreased upon titration with Glue_10_–BA (0–7 μM) at 25 °C in Tris–HCl buffer (50 mM Tris–HCl, 10 mM CaCl_2_, pH 8.0). According to the reported method,^27,28^ we estimated the association constant (*K*_assoc_) of Glue_10_–BA with trypsin to be 5.5 × 10^5^ M^–1^ by fitting the fractions of bound trypsin (Fig. 3, red) to the Hill equation.^27,28^ As expected, Glue_29_–BA bearing a larger number (29) of Gu^+^ pendants exhibited a significantly higher *K*_assoc_ value of 3.2 × 10^6^ M^–1^ (Fig. 3, brown and S10b†), reflecting an important role of multivalency. In sharp contrast, when TEG–BA without the glue moiety was used in the titration, the CD spectral change of trypsin was too small to detect unless the concentration range of TEG–BA for the titration was extended to 200 μM (Fig. 3, blue and S10a†). Accordingly, the *K*_assoc_ value was estimated to be 1.6 × 10^4^ M^–1^, which is 35- and 200-fold lower than those observed for Glue_10_–BA and Glue_29_–BA, respectively. We also found that Glue_10_–Ph without BA (Fig. 1) binds to trypsin (Fig. S9†) with a *K*_assoc_ value (2.8 × 10^5^ M^–1^; Fig. 3, green and S11a†) that is comparable to that of Glue_10_–BA, indicating that the glue moiety predominantly contributes to the binding affinity of Glue_10_–BA. Notably, the *K*_assoc_ value of ^*m*^Glue_27_–BA containing 27 Gu^+^ units along the main chain (8.2 × 10^4^ M^–1^; Fig. 3, purple and S11b†) was 40-fold lower than that of Glue_29_–BA possessing an almost comparable number of Gu^+^ pendants, and even 6.7-fold lower than that of Glue_10_–BA. As previously reported,^12^ the poor binding behaviour of ^*m*^Glue_27_–BA is most likely due to a presumably small conformational flexibility of its in-chain Gu^+^ units compared with that of the Gu^+^ units at the side-chain termini in Glue_*n*_–BA.

**Fig. 4:**
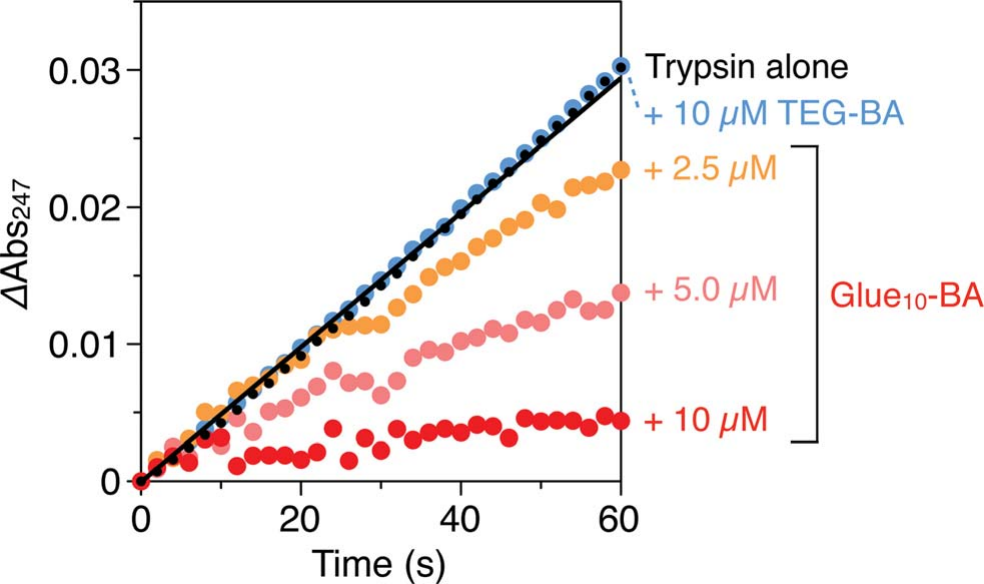
Absorption spectral changes at 247 nm of Tris–HCl buffer (50 mM Tris–HCl, 10 mM CaCl_2_, pH 8.0) solutions of a mixture of TAME (1 mM) and trypsin (20 nM) in the absence (black) and presence of 2.5 (orange), 5.0 (pink) and 10 μM (red) of Glue_10_–BA, and in the presence of 10 μM TEG–BA (blue).

**Fig. 5:**
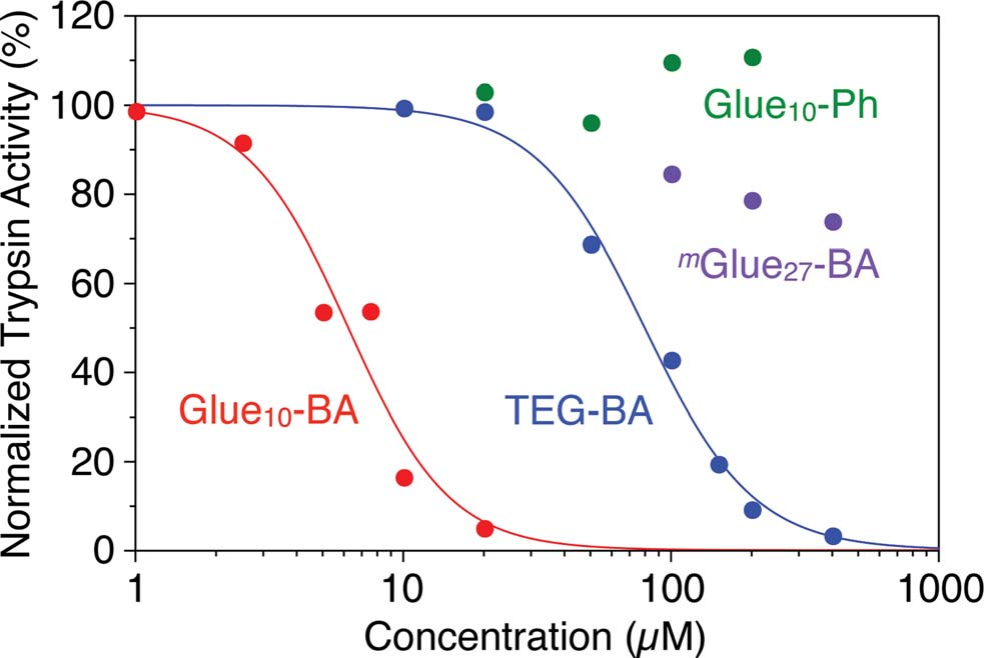
Hydrolytic activities of trypsin (20 nM), as estimated from the rates of absorption increase at 247 nm, normalized to that of untreated trypsin, at 25 °C in Tris–HCl buffer (50 mM Tris–HCl, 10 mM CaCl_2_, pH 8.0) containing TAME (1 mM) in the presence of TEG–BA (blue), Glue_10_–BA (red), Glue_10_–Ph (green) and ^*m*^Glue_27_–BA (purple).

Trypsin hydrolyses peptide linkages at the carboxyl side of lysine and arginine residues.^29^ As already described in Fig. 2a, this protease activity is inhibited by BA.^10^ Considering the exceptionally high affinity of Glue_29_–BA for trypsin, we expected that this BA-appended molecular glue might be the best inhibitor among those listed in Fig. 1. However, as observed by dynamic light scattering (DLS; Fig. S12†), trypsin/Glue_29_–BA, in contrast with other complexes such as trypsin/Glue_10_–BA and trypsin/Glue_10_–Ph, tends to form large aggregates (>200 nm), most likely due to the formation of physical crosslinks between its excessively long glue moiety and trypsin. Therefore, we conducted inhibitory assay experiments using Glue_10_–BA and ^*m*^Glue_27_–BA, along with Glue_10_–Ph and TEG–BA as references, but did not use Glue_29_–BA. Nevertheless, we found that upon conjugation with Glue_10_, BA’s inhibitory effect was considerably enhanced. As shown in Fig. 4 (black), when *N-p*-tosyl-l-arginine methyl ester (TAME, 1 mM) as a substrate was mixed with trypsin (20 nM) at 25 °C in Tris–HCl buffer (50 mM Tris–HCl, 10 mM CaCl_2_, pH 8.0), TAME was hydrolysed to *N-p*-tosyl-l-arginine (Fig. 2a), exhibiting an increase in its characteristic absorption at 247 nm.^10^ However, when 10 μM of Glue_10_–BA was added to the reaction system, the hydrolysis of TAME was considerably decelerated (Fig. 4, red). Although TEG–BA did not exhibit detectable inhibition at 10 μM (Fig. 4, blue), Glue_10_–BA explicitly inhibited the trypsin activity even at 2.5 μM (Fig. 4, orange). As shown in Fig. 5, the hydrolytic activity of trypsin was evaluated using the pseudo-first order reaction kinetics, and normalized to that of untreated trypsin (20 nM). The sigmoidal profile, obtained for the case with TEG–BA in Fig. 5 (blue), allowed estimation of the half-maximal inhibitory concentration (IC_50_) of TEG–BA as 79 μM. Notably, Glue_10_–BA exhibited a 13-fold greater inhibitory effect (IC_50_ = 6.2 μM; Fig. 5, red) than TEG–BA. In sharp contrast, when Glue_10_–Ph was used in place of Glue_10_–BA, no inhibition of the trypsin activity was observed (Fig. 5, green) even when [Glue_10_–Ph] was higher than 20 μM. Hence, the adhesion of the glue moiety does not hamper the enzymatic activity of trypsin, but primarily contributes to the stabilization of the BA/trypsin complex. As previously described, the binding affinity of ^*m*^Glue_27_–BA for trypsin is only 15% of that of Glue_10_–BA, but still 5-fold higher than that of TEG–BA. However, ^*m*^Glue_27_–BA exhibited a lower inhibitory effect than TEG–BA under identical conditions, and minimally inhibited the hydrolytic activity of trypsin (Fig. 5, purple). We presume that the poor conformational flexibility of the in-chain Gu^+^ units in the glue moiety hinders the ability of the conjugated BA terminus to properly block the substrate-binding site of trypsin. To rationalize the concept of blocker-appended molecular glues for pharmacological applications, this issue should be taken into consideration.

## Conclusions

Through a comparative inhibition study on the protease activity of trypsin using Glue_*n*_–BA, ^*m*^Glue_*n*_–BA and TEG–BA (Fig. 1) as potential trypsin inhibitors, we demonstrated that an active-site blocker such as BA efficiently inhibits the trypsin activity when its conjugated glue moiety (Glue_*n*_) can hold the blocker stably onto the active site through adhesion to a proximal oxyanionic region (Fig. 2b). Of particular interest is the obviously smaller inhibitory effect of ^*m*^Glue_27_–BA compared to TEG–BA, despite the fact that ^*m*^Glue_27_–BA has a 5-fold higher affinity than TEG–BA for trypsin. The incorporation of a mechanism to respond to biological or physical stimuli for controlling the operation of the blocker unit is an interesting subject worthy of further investigation.

## Methods

### Trypsin activity assay

To a Tris–HCl buffer (50 mM Tris–HCl, 10 mM CaCl_2_, pH 8.0) solution of trypsin (20 nM) was added a Tris–HCl buffer solution of TEG–BA, and the mixture was incubated at 25 °C for 1 min. Then, to the resultant solution was added a Tris–HCl buffer solution of *N-p*-tosyl-l-arginine methyl ester hydrochloride (TAME, final concentration 1 mM; Fig. 2a), and the absorption intensity at 247 nm (ref. 10) was traced over a period of 1 min. The trypsin activity was determined using pseudo-first order reaction kinetics and normalized to that of untreated trypsin. The trypsin activities in the presence of Glue_*n*_–BA, ^*m*^Glue_*n*_–BA and Glue_*n*_–Ph were likewise evaluated.
