# Supplementary material for: Molecular glues for manipulating enzymes: trypsin inhibition by benzamidine-conjugated molecular glues
Source: Chem Sci. 2015 Mar 18;6(5):2802–5. doi: 10.1039/c5sc00524h (PMC5489047; doi:10.1039/c5sc00524h)
Supplement: Supplementary file 1 [file SC-006-C5SC00524H-s001.pdf]

Electronic Supplementary Material (ESI) for Chemical Science.
This journal is © The Royal Society of Chemistry 2015

Electronic Supplementary Information

Molecular glues for manipulating enzymes: trypsin inhibition by benzamidine-conjugated molecular glues

Rina Mogaki,[a] Kou Okuro,*[a] and Takuzo Aida*[ab]

[a] *Department of Chemistry and Biotechnology, School of Engineering*

The University of Tokyo,

7-3-1 Hongo, Bunkyo-ku, Tokyo 113-8656, Japan

[b] *RIKEN Center for Emergent Matter Science*

2-1 Hirosawa, Wako, Saitama 351-0198, Japan

Table of Contents

1. **General**......S2
2. **Synthesis**......S3
3. **Static Light Scattering (SLS) Analysis**......S24
4. **Zeta Potential Measurements**......S26
5. **Circular Dichroism (CD) Spectral Titration**......S27
6. **Dynamic Light Scattering (DLS) Analysis**......S29
7. **References**......S30

1. General

^1H and ^{13}C NMR spectra were recorded on a JEOL type GSX-270 or JNM-ECA 500II spectrometer, where chemical shifts for ^1H NMR spectroscopy were determined with respect to non-deuterated solvent residues; $CHCl_3$ (δ 7.26), $CHD_2(CD_3)SO$ (δ 2.50), and HDO (δ 4.79), while those for ^{13}C NMR spectroscopy were determined with respect to $CHCl_3$ (δ 77.2) and $(CH_3)_2SO$ (δ 39.5). Matrix-assisted laser desorption/ionization time-of-flight mass (MALDI-TOF-MS) spectrometry was performed using α-cyano-4-hydroxy cinnamic acid (CCA) as a matrix on a Bruker Daltonics autoflex™ speed MALDI-TOF/TOF spectrometer. Normal phase column chromatography was carried out with Kanto Chemical silica gel 60 N (particle size 63–210 μm) or Merck alumina 90 standardized. Size-exclusion chromatography was carried out with GE Healthcare PD MidiTrap™ G-25. Electronic absorption spectra were recorded on a JASCO type V-670 spectrophotometer or a Thermo Scientific type NanoDrop ND-2000c spectrophotometer. Static light scattering (SLS) and dynamic light scattering (DLS) analyses were performed using a Malvern model Zetasizer μV particle size analyzer equipped with an 830 nm laser light source. Zeta potential measurements were performed using a Malvern model Zetasizer Nano ZS particle size analyzer equipped with a 633 nm He-Ne laser light source. Circular dichroism (CD) spectra were recorded on a JASCO type J-820 spectropolarimeter.

Unless otherwise noted, reagents and solvents were used as received from commercial sources without further purification. Tris-HCl (1.0 M) buffer solution (pH 8.0), hydrochloric acid (0.1 M), and calcium chloride solution (0.1 M) were purchased from Nacalai Tesque. *N*-*p*-tosyl-L-arginine methyl ester hydrochloride was purchased from TCI. Trypsin from porcine pancreas was purchased from Wako.

2. Synthesis

2-1. Synthesis of TEG-BA

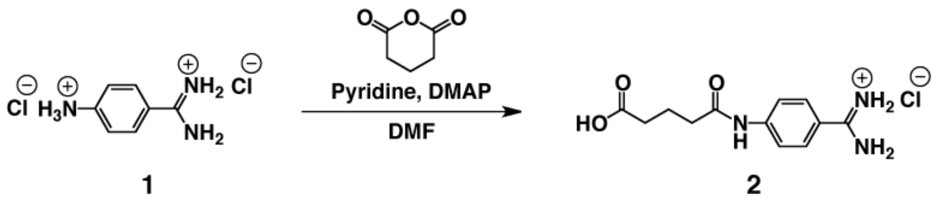

Compound 2. To a DMF (10 mL) solution of **1** (2.50 g, 12.0 mmol) were successively added pyridine (10 mL), *N*,*N*-dimethylaminopyridine (DMAP, 0.15 g, 1.23 mmol), and glutaric anhydride (1.37 g, 12.0 mmol), and the mixture was stirred for 30 min at 100 °C. Then, the resultant suspension was filtered, and an insoluble fraction isolated was washed successively with water (5 mL), MeCN (5 mL), and Et_2O (5 mL). The residue was suspended in Et_2O (10 mL), and then a 1,4-dioxane (10 mL) solution of HCl (4 M) was added to the suspension. After being stirred for 1 h at room temperature, the resultant suspension was filtered to allow isolation of **2** as white powder (1.82 g, 53%). ^{1}H NMR (500 MHz; DMSO-d_6; ppm): δ 1.78–1.84 (m, 2H; $CH_2CH_2CH_2$), 2.28 (t, J = 7.3 Hz, 2H; $CH_2CH_2CO_2H$), 2.42 (t, J = 7.5 Hz, 2H; CH_2CH_2CONH), 7.80 (s, 4H; ArH), 8.91 (s, 2H; $C(NH_2)N^+H_2$), 9.21 (s, 2H; $C(NH_2)N^+H_2$), 10.45 (s, 1H; CONH), 12.13 (br, 1H; CO_2H). ^{13}C NMR (126 MHz; DMSO-d_6; ppm): δ 20.2, 32.9, 35.5, 118.5, 121.5, 129.2, 144.3, 164.7, 171.6, 174.1. MALDI-TOF-MS (CCA): m/z found: 250.04 ([M – Cl^-] calcd: 250.12).

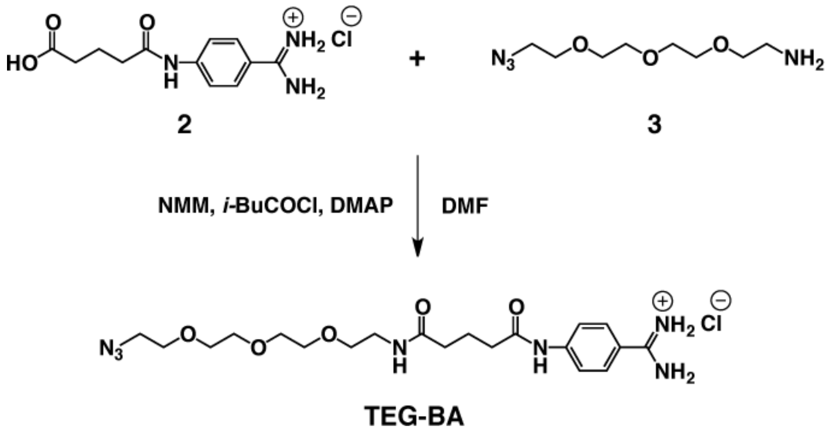

TEG-BA. To a DMF (28 mL) solution of **2** (0.70 g 2.18 mmol) were successively added *N*-methylmorpholine (NMM, 304 μL, 2.77 mmol) and isovaleryl chloride (*i*-BuCOCl, 325 μL, 2.67 mmol), and the mixture was stirred for 5 min at room temperature. Then, a DMF (14 mL) solution of a mixture of **3** (549 mg, 2.52 mmol), NMM (0.56 mL, 5.09 mmol), and *N*,*N*-dimethylaminopyridine (DMAP, 15 mg, 0.12 mmol) was added to the reaction mixture, and the resultant solution was stirred for 2 h at room temperature. Then, Et_2O (25 mL) was added to the reaction mixture, and an insoluble fraction was filtered off. The filtrate was evaporated to dryness under reduced pressure, and the residue was dissolved in MeOH and then reprecipitated with CH_2Cl_2. The precipitate was collected, dissolved in MeOH, and then reprecipitated with benzene. The precipitate was dissolved in $CHCl_3$ and reprecipitated twice with benzene. The precipitate was dissolved in an aqueous (3 mL) solution of HCl (1 M), and the resultant solution was stirred for 2 h at room temperature. The reaction mixture was washed with Et_2O (10 mL × 5) followed by AcOEt (10 mL × 3). An aqueous extract separated was evaporated to dryness under reduced pressure, affording **TEG-BA** as yellow solid (398 mg, 36%). ^{1}H NMR (500 MHz; D_2O; ppm): δ 1.95–2.01 (m, 2H; $CH_2CH_2CH_2$), 2.33 (t, J = 7.5 Hz, 2H; $CH_2CH_2CONHCH_2$), 2.48 (t, J = 7.3 Hz, 2H; $CH_2CH_2CONHAr$), 3.35 (t, J = 5.3 Hz, 2H; $CH_2CONHCH_2CH_2$), 3.43 (t, J = 4.8 Hz, 2H; CH_2N_3), 3.58 (t, J = 5.3 Hz, 2H; $CONHCH_2CH_2OCH_2$), 3.62–3.67 (m, 10H; OCH_2), 7.68 (d, J = 9.0 Hz, 2H; 2,6-ArH), 7.78 (d, J = 9.0 Hz, 2H; 3,5-ArH). ^{13}C NMR (126 MHz; DMSO-d_6; ppm): δ 21.0, 34.4, 35.8, 38.5, 50.0, 69.1, 69.3, 69.6, 69.7, 69.8, 118.4, 121.5, 129.2, 144.3, 164.7, 171.7, 171.8. MALDI-TOF-MS (CCA): *m/z* found: 472.25 ([M – HCl + Na^+] calcd: 472.23), 450.25 ([M – Cl^-] calcd: 450.25).

2-2. Synthesis of Glue$_n$-BA

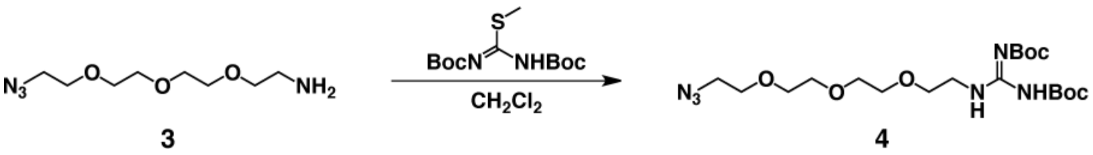

Compound 4. A CH_2Cl_2 (5 mL) solution of a mixture of **3** (1.00 g, 4.59 mmol) and 1,3-bis(*t*-butoxycarbonyl)-2-methylisothiourea (1.33 g, 4.59 mmol) was stirred for 4 h at room temperature. Then, the reaction mixture was diluted with AcOEt (45 mL) and washed with water (50 mL × 3). An organic extract separated was dried over Na_2SO_4 and filtered off from an insoluble fraction. The filtrate was evaporated to dryness under reduced pressure, and the residue was chromatographed on silica gel with AcOEt/hexane (1/4 to 2/1) as an eluent to allow isolation of **4** as colorless oil (1.72 g, 81%). ^{1}H NMR (500 MHz; $CDCl_3$; ppm): δ 1.49 (s, 9H; $C(CH_3)_3$), 1.50 (s, 9H; $C(CH_3)_3$), 3.39 (t, J = 5.0 Hz, 2H; CH_2N_3), 3.59–3.71 (m, 14H; OCH_2), 8.62 (br, 1H; N*H*CH_2), 11.48 (br, 1H; $NHCO_2$). ^{13}C NMR (126 MHz; $CDCl_3$; ppm): δ 28.2, 28.4, 40.8, 50.8, 69.5, 70.2, 70.6, 70.8, 70.9, 79.4, 83.1, 153.1, 156.4, 163.7. MALDI-TOF-MS (CCA): *m/z* found: 261.12 ([M – 2Boc + H$^+$] calcd: 261.17).

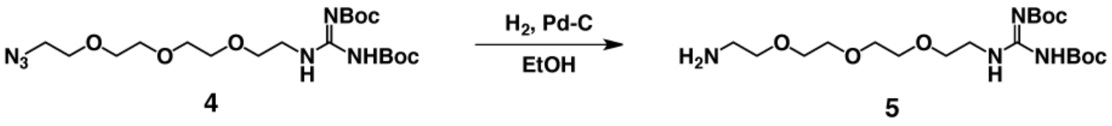

Compound 5. To an EtOH (5 mL) solution of **4** (1.00 g, 2.17 mmol) was added 10% palladium on carbon (52 mg), and the suspension was bubbled with H_2 for 18 h at room temperature. Then, the reaction mixture was filtered off with celite from an insoluble catalyst residue, and the filtrate was evaporated to dryness under reduced pressure. The residue was dissolved in EtOH (6 mL), and 10% palladium on carbon (52 mg) was added to the solution. The suspension was bubbled overnight with H_2 at room temperature. Then, the reaction mixture was filtered off with celite from an insoluble catalyst residue, and the filtrate was evaporated to dryness under reduced pressure. The residue was chromatographed on alumina with $CHCl_3$/MeOH (1/0 to 0/1) as an eluent to allow isolation of **5** as dark oil (706 mg, 75%). ^{1}H NMR (500 MHz; $CDCl_3$; ppm): δ 1.55 (t, J = 12 Hz, 18H; $C(CH_3)_3$), 3.10–4.30 (m, 16H; OCH_2), 8.55 (br, 1H; N*H*CH_2), 9.76 (br, 1H; CH_2N*H*$_2$), 11.46,

(br, 1H; $NHCO_2$). ^{13}C NMR (126 MHz; $CDCl_3$; ppm): δ 28.2, 28.5, 40.9, 69.5, 70.2, 70.4, 70.6, 70.7, 79.8, 83.3, 153.2, 156.7, 163.5. MALDI-TOF-MS (CCA): *m/z* found: 235.11 ([M – 2Boc + H^+] calcd: 235.18).

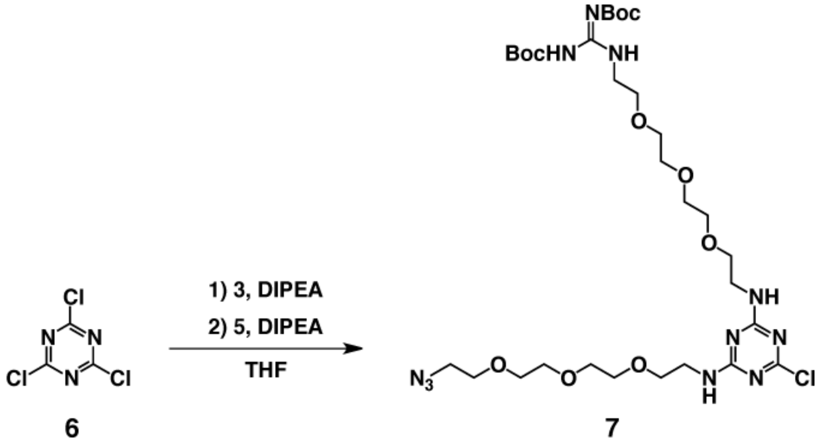

Compound 7. To a THF (3.4 mL) solution of a mixture of **6** (286 mg, 1.55 mmol) and diisopropylethylamine (DIPEA, 0.81 mL, 4.65 mmol) was added a THF (0.41 mL) solution of **3** (339 mg, 1.55 mmol) at 0 °C, and the mixture was stirred for 3 h at 0 °C. Then, the reaction mixture was allowed to warm to room temperature, and a THF (1.25 mL) solution of **5** (338 mg, 0.778 mmol) was added to the mixture. After being stirred overnight at room temperature, a THF (0.7 mL) solution of **5** (169 mg, 0.389 mmol) was added to the reaction mixture, and the mixture was stirred for 1 h at room temperature. Then, DIPEA (0.5 mL) was added to the reaction mixture, and the mixture was stirred for 4 h at room temperature. An insoluble fraction was filtered off, and then the filtrate was evaporated to dryness under reduced pressure. The residue was chromatographed on silica gel with AcOEt/hexane (1/4 to 1/0) as an eluent to allow isolation of **7** as white solid (633 mg, 53%). ^1H NMR (500 MHz; $CDCl_3$; ppm): δ 1.49 (s, 9H; $C(CH_3)_3$), 1.50 (s, 9H; $C(CH_3)_3$), 3.40 (t, J = 5.0 Hz, 2H; CH_2N_3), 5.63–5.88 (m, 2H; ArNH), 8.61 (br, 1H; N*H*CH_2), 11.5 (br, 1H; $NHCO_2$). ^{13}C NMR (126 MHz; $CDCl_3$; ppm): δ 28.2, 28.4, 40.7, 40.8, 50.8, 69.5, 70.2, 70.5, 70.6, 70.7, 70.8, 79.4, 83.0, 153.1, 156.4, 163.7, 163.9. MALDI-TOF-MS (CCA): *m/z* found: 564.31 ([M – 2Boc + H^+] calcd: 564.28).

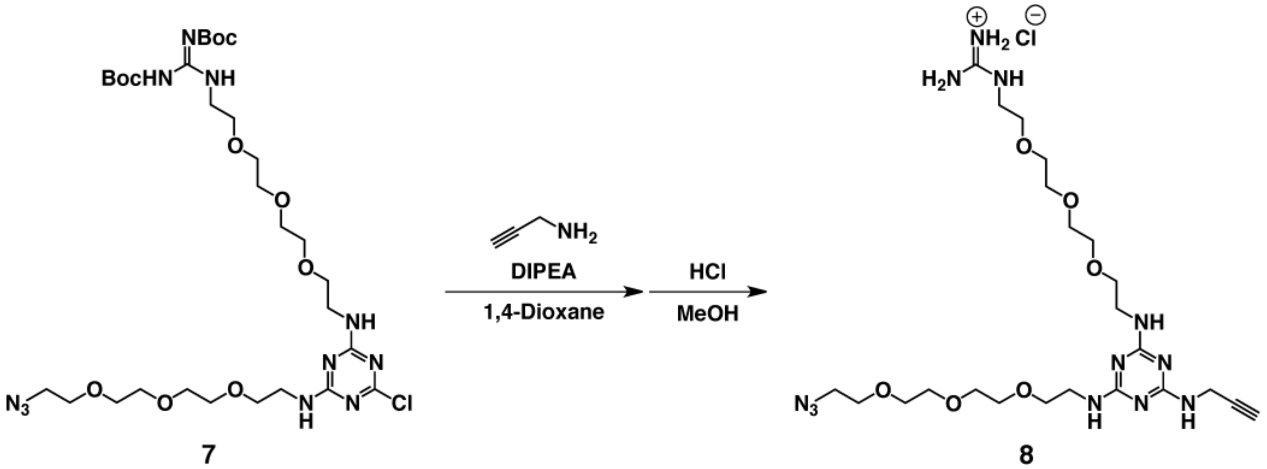

Compound 8. To a 1,4-dioxane (2.2 mL) solution of **7** (328 mg, 0.429 mmol) were successively added propargylamine (275 μL, 4.29 mmol) and diisopropylethylamine (DIPEA, 740 μL, 4.29 mmol), and the mixture was bubbled with argon for 1 min and stirred overnight at 80 °C. Then, an insoluble fraction was filtered off, and the filtrate was evaporated to dryness under reduced pressure. The residue was chromatographed on silica gel with AcOEt/hexane (2/1 to 1/0) and then AcOEt/MeOH (9/1) as eluents, and a fraction thus obtained was evaporated to dryness under reduced pressure. Then, MeOH (2.5 mL) and hydrochloric acid (12 M, 2.5 mL) were successively added to the residue, and the resultant mixture was stirred for 6 h at room temperature. The reaction mixture was washed with Et_2O (5 mL × 2) followed by AcOEt (5 mL × 2), and an aqueous extract separated was evaporated to dryness under reduced pressure, affording **8** as brown oil (134 mg, 50%). ^{1}H NMR (500 MHz; D_2O; ppm): δ 2.63 (br, 1H; $CH_2CC\mathit{H}$), 3.41–3.52 (m, 4H; $C\mathit{H}_2N_3$, $C\mathit{H}_2$-guanidine), 3.61–3.79 (br, 28H; OCH_2), 4.23 (br, 2H; $C\mathit{H}_2CCH$). ^{13}C NMR (126 MHz; DMSO-d_6; ppm): δ 30.0, 41.1, 50.0, 67.9, 68.3, 69.3, 69.7, 69.8, 69.8, 73.6, 80.4, 153.6, 154.0, 154.7. MALDI-TOF-MS (CCA): *m/z* found: 583.46 ([M – Cl^-] calcd: 583.34).

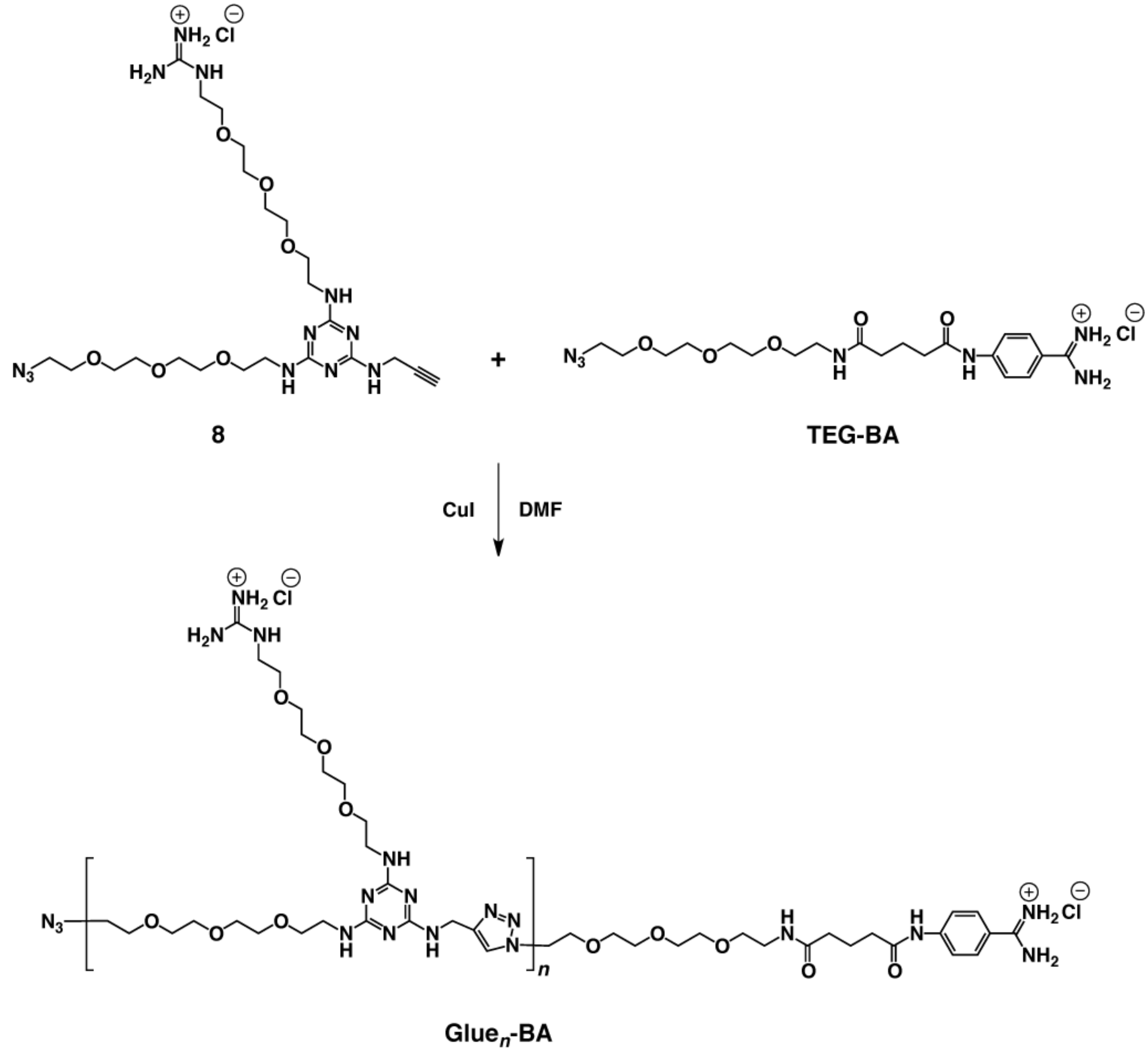

Glue$_n$-BA. To a DMF (65 μL) solution of a mixture of **8** (39 mg, 64 μmol) and **TEG-BA** (1.2 mg, 2.4 μmol) was added copper(I) iodide (CuI, 10 mg, 53 μmol), and the mixture was stirred vigorously for 10 min at room temperature. Then, Et_2O (300 μL) was added to the reaction mixture, and an insoluble fraction separated was collected and dissolved in DMF (100 μL), and then reprecipitated twice with Et_2O. A precipitate was collected and dissolved in a 1,4-dioxane (300 μL) solution of HCl (4 M), and MeCN (300 μL) was added to the solution. The resultant solution was subjected to reprecipitation with Et_2O, and a precipitate formed was collected and dissolved in hydrochloric acid (4 M, 300 μL). This solution was subjected to size-exclusion chromatography on Sephadex G-25 using water as an eluent to allow separation of **Glue$_n$-BA**

into 2 fractions (fractions 4 (6.4 mg) and 5 (8.6 mg) in Fig. S1) in terms of molecular weight.

Fraction 4: ^{1}H NMR (Fig. S2, 500 MHz; D_2O; ppm): δ 1.90–2.02 (m, 2H; $CH_2CH_2CH_2$), 2.27–2.37 (m, 2H; $CH_2C\mathit{H}_2CONHCH_2$), 2.41–2.53 (m, 2H; $CH_2C\mathit{H}_2CONHAr$), 3.28–3.86 (m; OCH_2), 3.87–4.02 (m; triazole-$CH_2C\mathit{H}_2$), 4.50–4.72 (m; triazole-$C\mathit{H}_2CH_2$), 7.63–7.71 (br, 2H; 2,6-ArH), 7.95–8.12 (m; triazole-H).

Fraction 5: ^{1}H NMR (Fig. S3, 500 MHz; D_2O; ppm): δ 1.89–2.00 (br, 2H; $CH_2C\mathit{H}_2CH_2$), 2.27–2.35 (br, 2H; $CH_2C\mathit{H}_2CONHCH_2$), 2.41–2.51 (br, 2H; $CH_2C\mathit{H}_2CONHAr$), 3.29–3.85 (m; OCH_2), 3.85–4.04 (m; triazole-$CH_2C\mathit{H}_2$), 4.52–4.68 (m; triazole-$C\mathit{H}_2CH_2$), 7.61–7.70 (m, 2H; 2,6-ArH), 7.72–7.79 (m, 2H; 3,5-ArH), 7.96–8.17 (br; triazole-H).

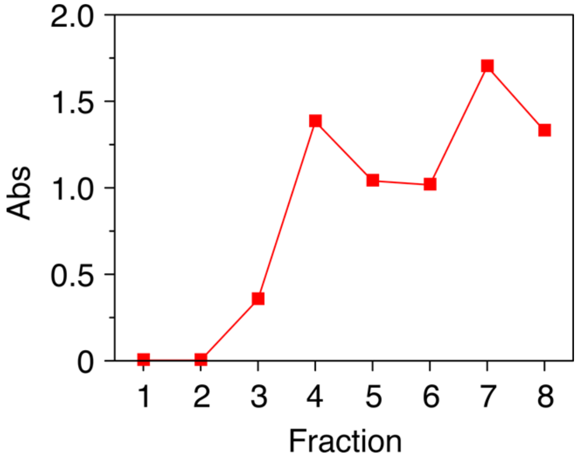

Fig. S1 SEC trace of Glue$_n$-BA monitored at 280 nm. The sample was fractionated into 8 fractions (500 µL each), and fractions 4 and 5 were isolated and identified as Glue$_{29}$-BA and Glue$_{10}$-BA (n in Glue$_n$-BA represents an average number of the repeating units), respectively, by ^{1}H NMR (Fig. S2 and S3).

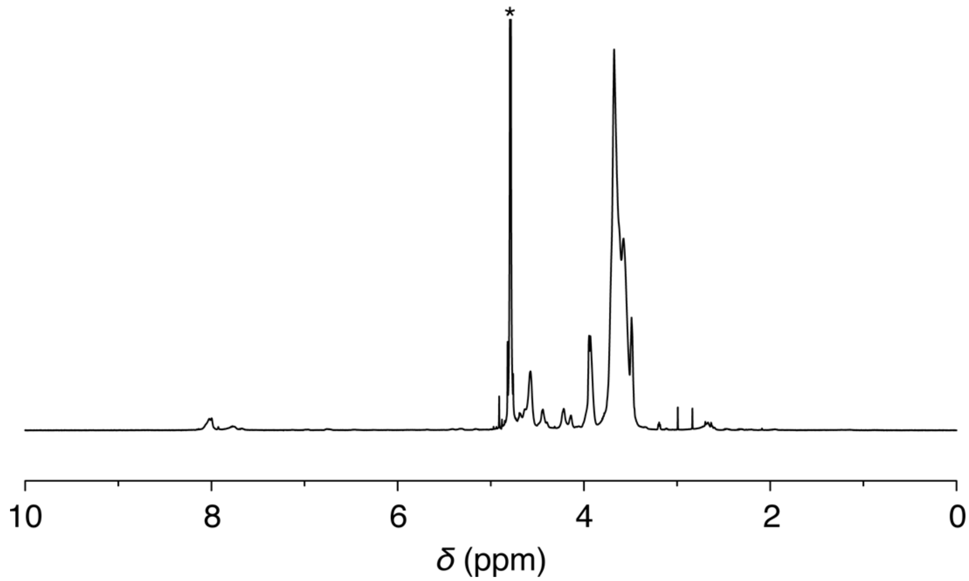

Fig. S2 ^{1}H NMR spectrum (500 MHz) of a D_2O solution of Glue$_{29}$-BA (Fig. S1, fraction 4) at 21 °C. An asterisked signal at δ 4.79 ppm is due to water. The average number of the repeating units was estimated as 29 from the intensity ratio of signals at 2.27–2.37 ppm (2H; $CH_2CH_2CONHCH_2$) to 7.95–8.12 ppm (triazole-H).

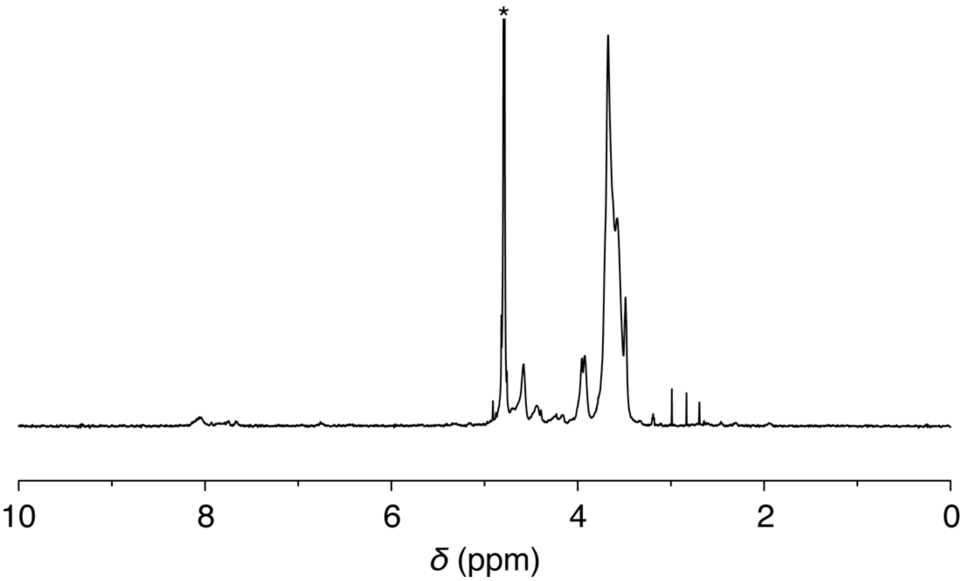

Fig. S3 ^{1}H NMR spectrum (500 MHz) of a D_2O solution of $Glue_{10}$-BA (Fig. S1, fraction 5) at 21 °C. An asterisked signal at δ 4.79 ppm is due to water. The average number of the repeating units was estimated as 10 from the intensity ratio of signals at 2.27–2.35 ppm (2H; $CH_2CH_2CONHCH_2$) to 7.96–8.17 ppm (triazole-H).

2-3. Synthesis of ^{m}Glue$_{n}$-BA

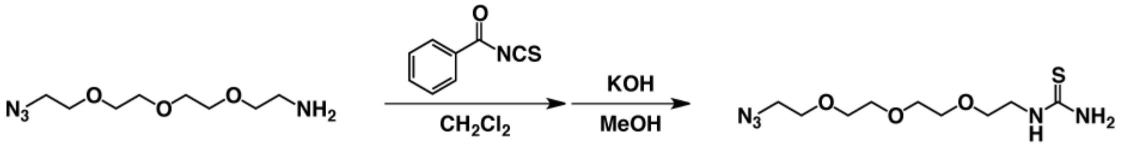

3 9

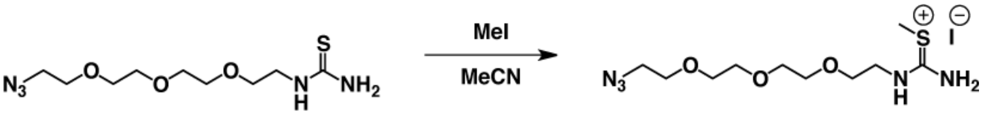

9 10

Compound 9. To a dry CH_2Cl_2 (75 mL) solution of **3** (1.92 g, 8.4 mmol) was added benzoyl isothiocyanate (1.50 mL, 11.2 mmol) at 0 °C under argon, and the mixture was stirred for 3 h at room temperature. Then, the reaction mixture was evaporated to dryness under reduced pressure, and the residue was dissolved in MeOH (40 mL). After the addition of KOH (1.80 g, 32.1 mmol), the MeOH solution was stirred for 40 min at room temperature, and then water (75 mL) was added to the reaction mixture. After being stirred overnight at room temperature, the reaction mixture was extracted with CH_2Cl_2 (25 mL) and washed with aqueous KOH (1 M, 25 mL) followed by brine (25 mL). An organic extract separated was dried over Na_2SO_4 and filtered off from an insoluble fraction. The filtrate was evaporated to dryness under reduced pressure, and the residue was chromatographed on silica gel with AcOEt/CH_2Cl_2 (1/1 to 1/0) as an eluent to allow isolation of **9** as pale yellow oil (1.68 g, 72%). ^{1}H NMR (270 MHz; $CDCl_3$; ppm): δ 3.44 (br, 2H; CH_2N_3), 3.55–3.73 (m, 12H; OCH_2), 3.78 (br, 2H; CH_2NH). ^{13}C NMR (67.8 MHz; $CDCl_3$; ppm): δ 45.3, 50.7, 69.5, 69.8, 70.0, 70.3, 70.4, 70.6, 71.3, 183.6. MALDI-TOF-MS (CCA): *m/z* found: 316.00 ([M + K^+] calcd: 316.08), 300.01 ([M + Na^+] calcd: 300.11).

Compound 10. To a MeCN (40 mL) solution of **9** (1.50 g, 5.41 mmol) was added MeI (10 mL), and the mixture was stirred for 1 h at 40 °C. Then, the reaction mixture was evaporated to dryness under reduced pressure, and the residue was dissolved in $CHCl_3$ and reprecipitated with hexane to allow isolation of **10** as yellow oil (2.18 g, 96%). ^{1}H NMR (270 MHz; $CDCl_3$; ppm): δ 2.92 (s, 3H; S^+CH_3), 3.50–3.73 (m, 12H; OCH_2, CH_2N_3), 3.83 (br, 4H; $CH_2CH_2NHCS^+$, $CH_2CH_2NHCS^+$). MALDI-TOF-MS (CCA): *m/z* found: 330.04 ([M – HI + K^+] calcd: 330.10),

314.06 ([M – HI + Na$^+$] calcd: 314.13), 292.07 ([M – I$^-$] calcd: 292.14).

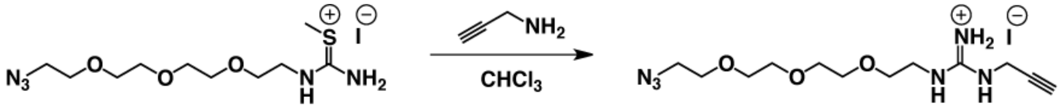

10 11

Compound 11. To a $CHCl_3$ (50 mL) solution of **10** (2.12 g, 5.1 mmol) was added propargylamine (1.50 g, 27.2 mmol), and the mixture was stirred for 4 h at room temperature. Then, the reaction mixture was evaporated to dryness under reduced pressure, and the residue was dissolved in $CHCl_3$ and reprecipitated with hexane to allow isolation of **11** as orange oil (2.01 g, 93%). ^{1}H NMR (270 MHz; $CDCl_3$; ppm): δ 2.46 (t, J = 4.8 Hz, 1H; CH_2CCH), 3.46 (t, J = 9.0 Hz, 4H; CH_2N_3, CH_2CH_2-guanidine), 3.57–3.77 (m, 12H; OCH_2), 4.08 (br, 2H; CH_2CCH). ^{13}C NMR (67.8 MHz; $CDCl_3$; ppm): δ 32.0, 43.2, 50.9, 69.3, 69.9, 67.0, 70.1, 70.3, 70.8, 71.5, 74.7, 158.2. MALDI-TOF-MS (CCA): m/z found: 337.11 ([M – HI + K$^+$] calcd: 337.14), 321.13 ([M – HI + Na$^+$] calcd: 321.16), 299.12 ([M – I$^-$] calcd: 299.18).

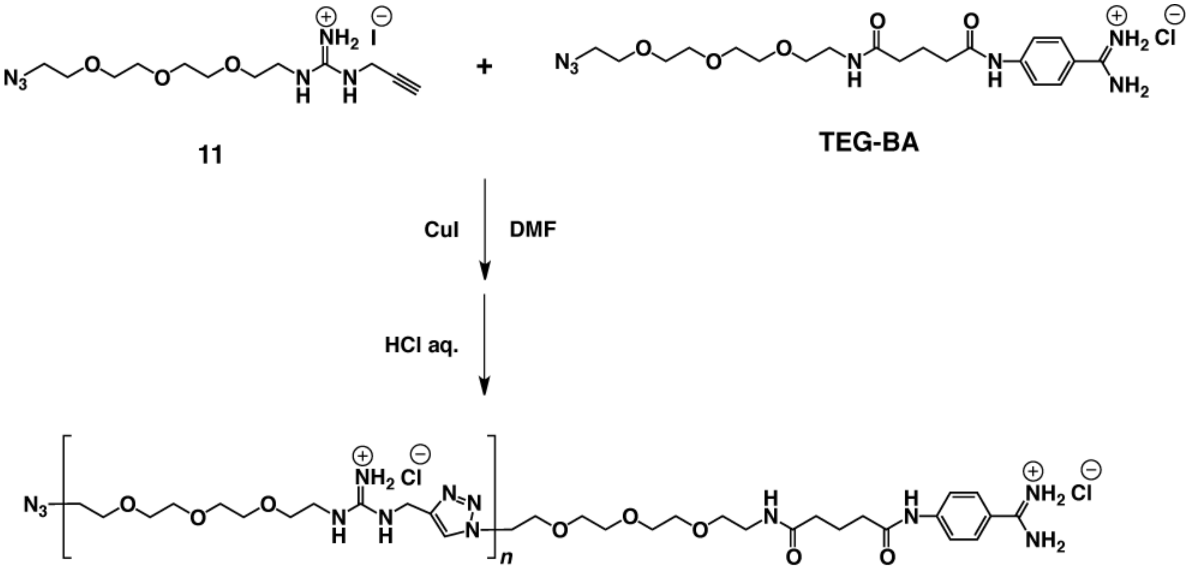

^{m}Glue$_n$-BA

^{m}Glue$_n$-BA. To a DMF (94 µL) solution of a mixture of **11** (41 mg, 94 µmol) and **TEG-BA** (2.3 mg, 4.7 µmol) was added copper(I) iodide (CuI, 10 mg, 53 µmol), and the mixture was stirred vigorously for 10 min at room temperature. Then, Et_2O (300 µL) was added to the reaction

mixture, and an insoluble fraction separated was dissolved in DMF (100 μL) and reprecipitated three times with Et_2O. The precipitate was dissolved in a 1,4-dioxane solution (300 μL) of HCl (4 M), and DMF (100 μL) was added to the solution. The resultant mixture was reprecipitated three times with MeCN, and a precipitate was collected and dissolved in hydrochloric acid (4 M, 300 μL). This solution was subjected to size-exclusion chromatography on Sephadex G-25 with water as an eluent to allow separation of **^{m}Glue$_{n}$-BA** (fraction 4, Fig. S4) as yellow solid (9.6 mg). ^{1}H NMR (500 MHz; D_2O; ppm): δ 1.91–2.00 (m, 2H; $CH_2CH_2CH_2$), 2.31 (t, J = 7.5 Hz, 2H; $CH_2CH_2CONHCH_2$), 2.47 (t, J = 7.3 Hz, 2H; $CH_2CH_2CONHAr$), 3.31–3.48 (m; CH_2CH_2-guanidine), 3.49–3.83 (m; OCH_2), 3.85–4.03 (m; triazole-CH_2CH_2O), 4.48–4.64 (m; guanidine-CH_2-triazole, triazole-CH_2CH_2O), 7.66 (d, J = 8.5 Hz, 2H; 2,6-ArH), 7.76 (d, J = 8.5 Hz, 2H; 3,5-ArH), 8.02 (s; triazole-H).

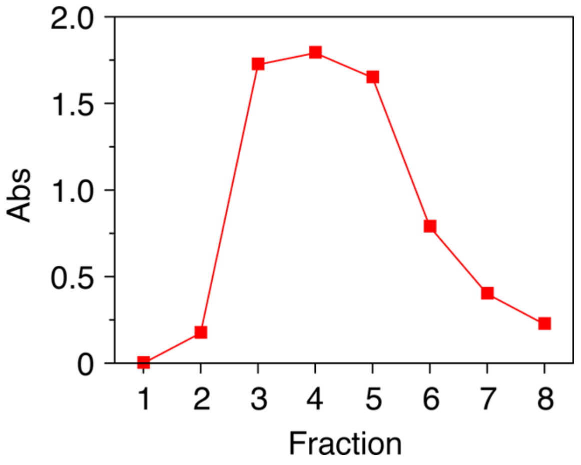

Fig. S4 SEC trace of ^{m}Glue$_{n}$-BA monitored at 280 nm. The sample was fractionated into 8 fractions (500 μL each), where fraction 4 was collected and identified by ^{1}H NMR as ^{m}Glue$_{27}$-BA (n in ^{m}Glue$_{n}$-BA represents an average number of the repeating units) (Fig. S5).

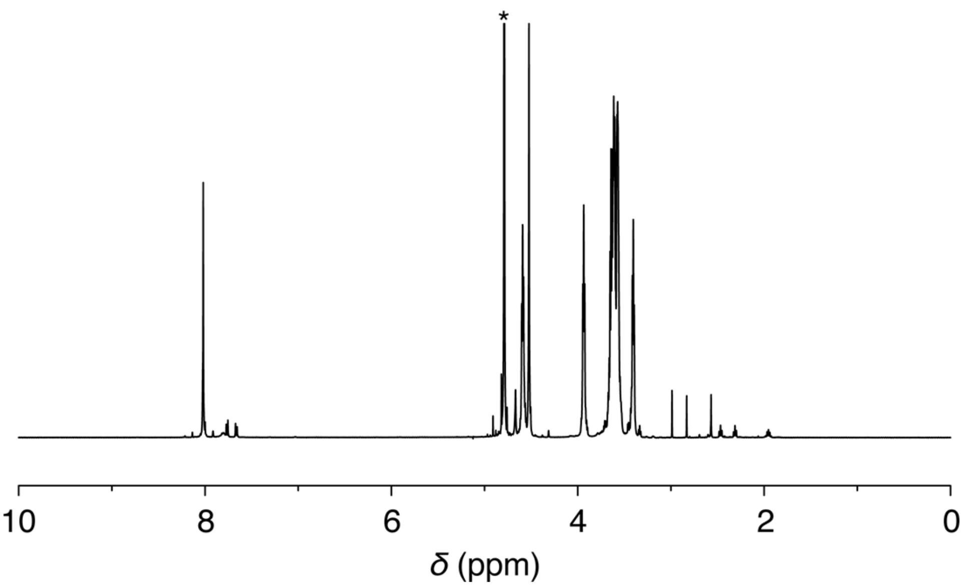

Fig. S5 ^{1}H NMR spectrum (500 MHz) of a D_2O solution of ^{m}Glue$_{27}$-BA (Fig. S4, fraction 4) at 21 °C. An asterisked signal at δ 4.79 ppm is due to water. The average number of the repeating units was estimated as 27 from the intensity ratio of signals at 2.31 ppm (2H; $CH_2C\mathit{H}_2CONHCH_2$) to 8.02 ppm (triazole-H).

2-4. Synthesis of Glue$_n$-Ph

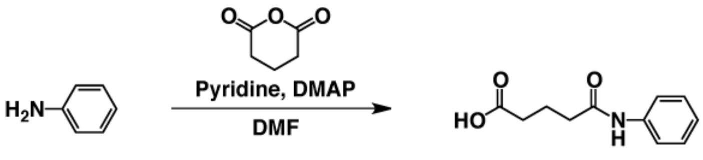

12 13

Compound 13. To a DMF (10 mL) solution of **12** (1.20 g, 12.9 mmol) were successively added pyridine (10 mL), *N*,*N*-dimethylaminopyridine (DMAP, 0.16 g, 1.29 μmol), and glutaric anhydride (1.47 g, 12.9 mmol), and the mixture was stirred for 40 min at 100 °C. Then, the reaction mixture was evaporated to dryness under reduced pressure, and the residue was dissolved in AcOEt (20 mL) and washed successively with saturated aqueous NH_4Cl (20 mL × 3), water (20 mL), and brine (20 mL). An organic extract separated was dried over Na_2SO_4 and filtered off from an insoluble fraction. The filtrate was evaporated to dryness under reduced pressure, and the residue was chromatographed on silica gel with AcOEt/hexane (2/1 to 1/0) and then AcOEt/MeOH (9/1) as eluents. The obtained fraction was recrystallized from CH_2Cl_2/EtOH (1/1) to allow isolation of **13** as white powder (377 mg, 14%). ^{1}H NMR (500 MHz; DMSO-d_6; ppm): δ 1.77–1.83 (m, 2H; $CH_2CH_2CH_2$), 2.27 (t, J = 7.0 Hz, 2H; $CH_2CH_2CO_2H$), 2.34 (t, J = 7.5 Hz, 2H; CH_2CH_2CONH), 7.01 (dd, J = 7.5 Hz, 1H; 4-ArH), 7.26–7.30 (m, 2H; 3,5-ArH), 7.58 (d, J = 7.5 Hz, 2H; 2,6-ArNH), 9.88 (br, 1H; CONH), 12.07 (br, 1H; CO_2H). ^{13}C NMR (126 MHz; DMSO-d_6; ppm): δ 20.4, 33.0, 35.4, 119.0, 123.0, 128.7, 139.3, 170.7, 174.2. MALDI-TOF-MS (CCA): *m/z* found: 230.12 ([M + Na$^+$] calcd: 230.08).

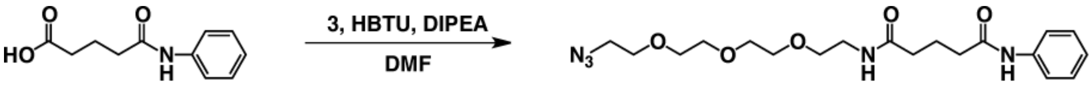

13 TEG-Ph

TEG-Ph. To a DMF (4 mL) solution of a mixture of **13** (151 mg, 0.72 mmol) and **3** (159 mg, 0.73 mmol) were successively added *o*-(benzotriazol-1-yl)-*N*,*N*,*N'*,*N'*-tetramethyluronium hexafluorophosphate (HBTU, 288 mg, 0.76 mmol) and diisopropylethylamine (DIPEA, 125 μL, 0.72 mmol), and the mixture was stirred overnight at room temperature. Then, the reaction mixture was evaporated to dryness under reduced pressure. The residue was dissolved in AcOEt

(15 mL) and washed successively with saturated aqueous NH_4Cl (15 mL), saturated aqueous $NaHCO_3$ (15 mL), water (15 mL), and brine (15 mL). An organic extract separated was dried over Na_2SO_4 and filtered off from an insoluble fraction. The filtrate was evaporated to dryness under reduced pressure, and the residue was chromatographed on alumina with AcOEt/MeOH (1/0 to 99/1) as an eluent, where the main fraction was collected and evaporated to dryness under reduced pressure. The residue was dissolved in $CHCl_3$ and reprecipitated with hexane to allow isolation of **TEG-Ph** as white solid (203 mg, 69%). ^{1}H NMR (500 MHz; $CDCl_3$; ppm): δ 2.01–2.10 (m, 2H; $CH_2CH_2CH_2$), 2.32 (t, J = 6.8 Hz, 2H; $CH_2CH_2CONHCH_2$), 2.46 (m, J = 7.0 Hz, 2H; $CH_2CH_2CONHAr$), 3.35 (t, J = 5.3 Hz, 2H; $CH_2CONHCH_2CH_2$), 3.47 (dd, J = 5.3 Hz, 2H; CH_2N_3), 3.58 (t, J = 5.0 Hz, 2H; $CONHCH_2CH_2OCH_2$), 3.60–3.69 (m, 10H; OCH_2), 6.21 (br, 1H; CONHAr), 7.08 (dd, J = 7.3 Hz, 1H; 4-ArH), 7.31 (dd, J = 8.0 Hz, 2H; 3,5-ArH), 7.57 (d, J = 7.5 Hz, 2H; 2,6-ArH), 8.36 (br, 1H; $CONHCH_2$). ^{13}C NMR (126 MHz; $CDCl_3$; ppm): δ 22.1, 29.8, 35.0, 36.3, 39.4, 50.8, 69.8, 70.1, 70.4, 70.7, 70.8, 119.7, 124.1, 129.1, 138.4, 171.2, 173.0. MALDI-TOF-MS (CCA): m/z found: 446.31 ([M + K^+] calcd: 446.18), 430.33 ([M + Na^+] calcd: 430.21), 408.35 ([M + H^+] calcd: 408.22).

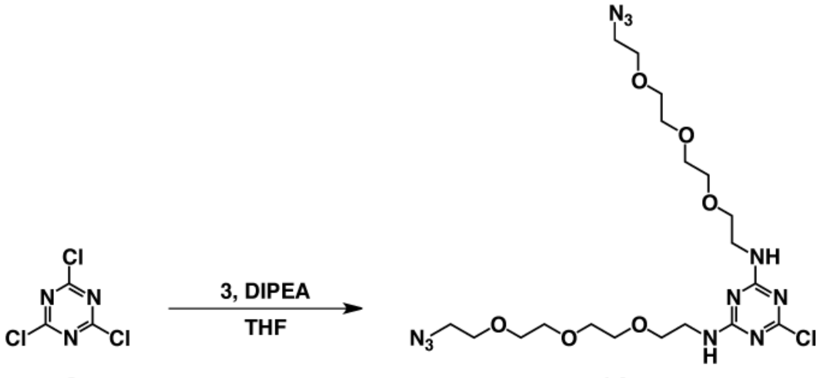

Compound 14. To a THF (5 mL) solution of **6** (0.42 g, 2.29 mmol) were successively added diisopropylethylamine (DIPEA, 1.20 mL, 6.87 mmol) and a THF (1.6 mL) solution of **3** (1.00 g, 4.58 mmol), and the mixture was stirred overnight at room temperature. Then, DIPEA (0.60 mL, 3.44 mmol) was added to the reaction mixture, and the resultant solution was stirred for 1 h at room temperature. An insoluble fraction was filtered off, and the filtrate was evaporated to dryness under reduced pressure. The residue was chromatographed on silica gel with AcOEt/hexane (1/2 to 1/0) and then AcOEt/MeOH (95/5) as eluents to allow isolation of **14** as white solid (992 mg, 79%). ^{1}H

NMR (500 MHz; $CDCl_3$; ppm): δ 3.40 (t, J = 5.0 Hz, 4H; CH_2N_3), 3.51–3.84 (m, 28H; OCH_2), 5.76–6.02 (br, 2H; ArNH). ^{13}C NMR (126 MHz; $CDCl_3$; ppm): δ 40.8, 50.8, 69.5, 69.8, 70.2, 70.5, 70.6, 70.8, 165.7. MALDI-TOF-MS (CCA): *m/z* found: 570.15 ([M + Na^+] calcd: 570.23), 548.16 ([M + H^+] calcd: 548.25).

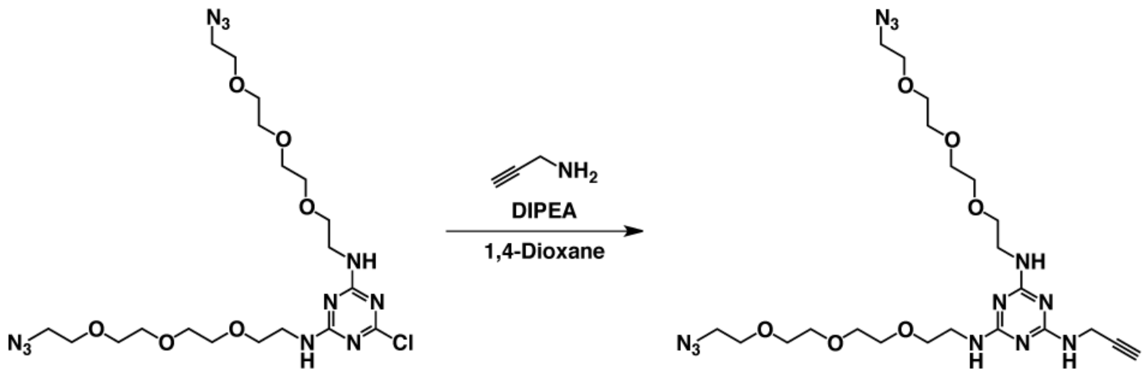

14 **15**

Compound 15. To a 1,4-dioxane (6.6 mL) solution of **14** (700 mg, 1.28 mmol) were successively added propargylamine (0.82 mL, 12.8 mmol) and diisopropylethylamine (DIPEA, 2.20 mL, 12.8 mmol), and the mixture was bubbled with argon for 1 min and stirred overnight at 80 °C. An insoluble fraction was filtered off, and the filtrate was evaporated to dryness under reduced pressure. The residue was chromatographed on silica gel with AcOEt/MeOH (1/0 to 9/1) as an eluent to allow isolation of **15** as brown oil (178 mg, 25%). ^1H NMR (500 MHz; $CDCl_3$; ppm): δ 2.20 (t, J = 2.5 Hz, 1H; $CH_2CC\mathit{H}$), 3.39 (t, 4H; CH_2N_3), 3.46–3.73 (m, 28H; OCH_2), 4.16 (br, 2H; $C\mathit{H_2}CCH$). ^{13}C NMR (126 MHz; $CDCl_3$; ppm): δ 30.4, 40.5, 50.8, 70.2, 70.3, 70.5, 70.8, 81.3, 166.1. MALDI-TOF-MS (CCA): *m/z* found: 605.50 ([M + K^+] calcd: 605.27), 589.52 ([M + Na^+] calcd: 589.29), 567.53 ([M + H^+] calcd: 567.31).

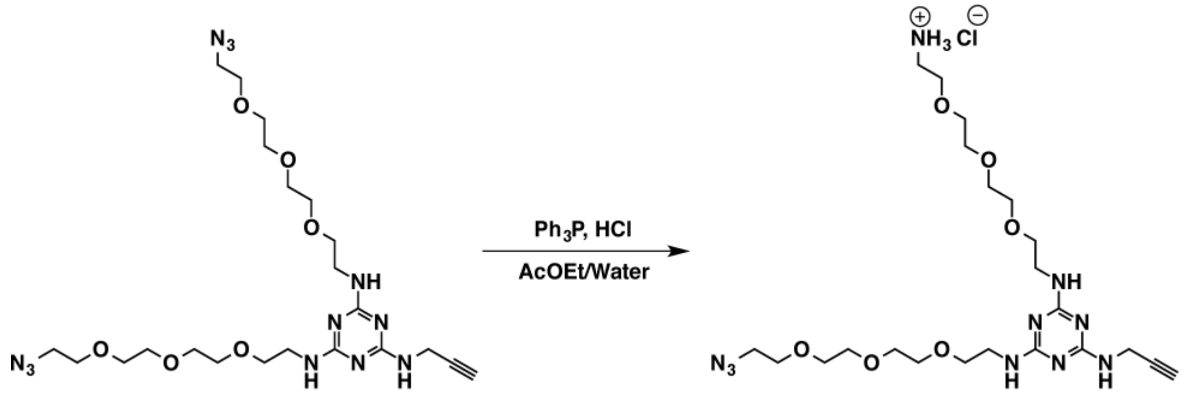

15 **16**

Compound 16. To an AcOEt (5.0 mL) solution of **15** (151 mg, 0.266 mmol) were successively added hydrochloric acid (1 M, 1.0 mL) and triphenylphosphine (84.6 mg, 0.323 mmol), and the mixture was stirred overnight at room temperature. Then, the reaction mixture was diluted with water (14 mL) and washed with AcOEt (20 mL × 3). An aqueous extract separated was evaporated to dryness under reduced pressure to give **16** as brown oil (165 mg, quant.). ^{1}H NMR (500 MHz; $CDCl_3$; ppm): δ 2.29 (s, 1H; CH_2CCH), 3.24 (br, 2H; $OCH_2CH_2N^+H_3$), 3.40 (br, 2H; CH_2N_3), 3.68 (br, 26H; OCH_2), 3.87 (br, 2H; $OCH_2CH_2N^+H_3$), 4.22 (br, 2H; CH_2CCH), 8.33 (br, 3H; $CH_2N^+H_3$). ^{13}C NMR (126 MHz; $CDCl_3$; ppm): δ 30.6, 40.0, 41.0, 50.8, 66.7, 69.0, 70.0, 70.1, 70.4, 70.6, 70.7, 70.8, 72.3, 155.2. MALDI-TOF-MS (CCA): *m/z* found: 579.51 ([M – HCl + K^+] calcd: 579.28), 541.54 ([M – Cl^-] calcd: 541.32).

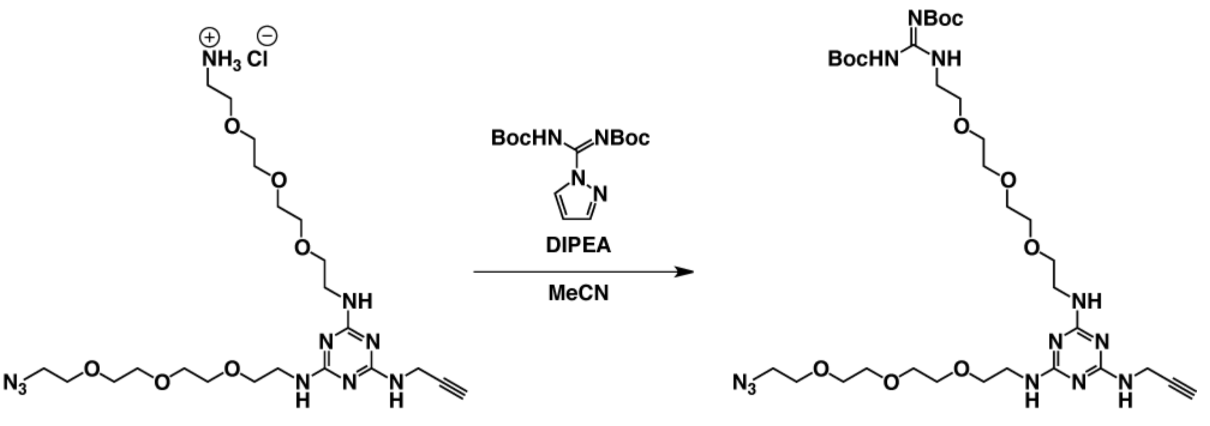

16 **17**

Compound 17. To a MeCN (2.5 mL) solution of a mixture of **16** (153 mg, 0.265 mmol) and *N*, *N'*-bis(*t*-boc)-1*H*-pyrazole-1-carboxamidine (123 mg, 0.398 mmol) was added diisopropylethylamine

(DIPEA, 91 µL, 0.530 mmol), and the mixture was stirred for 9 h at room temperature. Then, DIPEA (41 µL, 0.239 mmol) was again added, and the mixture was stirred for 12 h at room temperature. Then, the reaction mixture was evaporated to dryness under reduced pressure, and the residue was chromatographed on silica gel with AcOEt/MeOH (1/0 to 95/5) as an eluent to allow isolation of **17** as brown oil (99.1 mg, 48%). ^{1}H NMR (500 MHz; $CDCl_3$; ppm): δ 1.49 (s, 9H; $C(CH_3)_3$), 1.50 (s, 9H; $C(CH_3)_3$), 2.20 (t, J = 2.5 Hz, 1H; $CH_2CC\mathit{H}$), 3.40 (t, J = 5.0 Hz, 2H; CH_2N_3), 3.46–3.72 (m, 30H; OCH_2), 4.16 (br, 2H; $C\mathit{H}_2CCH$), 8.62 (br, 1H; $N\mathit{H}CH_2$), 11.47 (br, 1H; $NHCO_2$). ^{13}C NMR (126 MHz; $CDCl_3$; ppm): δ 28.2, 28.4, 30.5, 40.5, 40.8, 50.8, 69.5, 70.2, 70.3, 70.5, 70.6, 70.7, 70.8, 70.9, 79.4, 81.0, 83.1, 153.1, 156.4, 163.7, 166.0. MALDI-TOF-MS (CCA): *m/z* found: 583.60 ([M – 2Boc + H^+] calcd: 583.34).

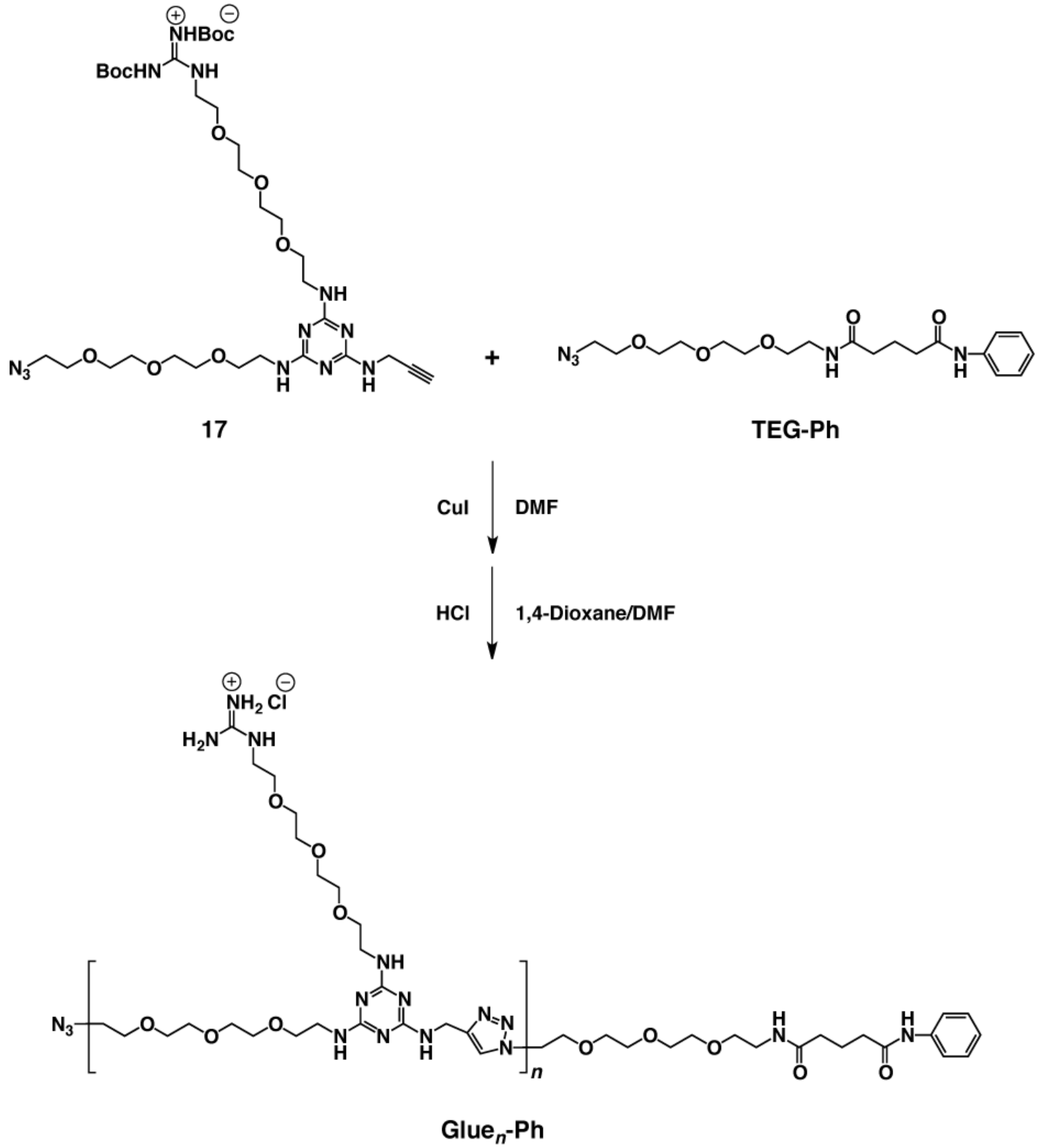

Glue$_n$-Ph. To a DMF (49 μL) solution of a mixture of **17** (38 mg, 49 μmol) and **TEG-Ph** (1.3 mg, 3.3 μmol) was added copper(I) iodide (10 mg, 53 μmol), and the mixture was stirred vigorously for 10 min at room temperature. Then, $CHCl_3$ (200 μL) was added to the resultant mixture and washed with water (300 μL × 5). An organic extract separated was dried over Na_2SO_4 and filtered off with celite from an insoluble fraction. The filtrate was evaporated to dryness under reduced pressure, and the residue was dissolved in a 1,4-dioxane/DMF (1.2 mL, v/v = 5/1) solution of HCl (3.3 M). After being stirred for 2 h at room temperature, Et_2O (1 mL)

was added to the resultant mixture, and a precipitate formed was collected and washed with Et_2O (1 mL). The precipitate was dissolved in DMF (100 μL) and reprecipitated with Et_2O. The precipitate was dissolved in hydrochloric acid (4 M, 300 μL), and the resultant solution was subjected to size-exclusion chromatography on Sephadex G-25 with water as an eluent to allow separation of **Glue*n*-Ph** (fraction 5, Fig. S6) as brown solid (7.4 mg). ^{1}H NMR (500 MHz; D_2O; ppm): δ 1.86–1.99 (m, 2H; $CH_2CH_2CH_2$), 2.24–2.36 (m, 2H; $CH_2CH_2CONHCH_2$), 2.36–2.46 (m, 2H; $CH_2CH_2CONHAr$), 3.27–3.39 (m; CH_2CH_2NH-triazine), 3.41–3.83 (m; OCH_2), 3.83–4.03 (triazole-CH_2CH_2), 7.20–7.43 (m, 5H; ArH), 7.94–8.15 (br; triazole-H).

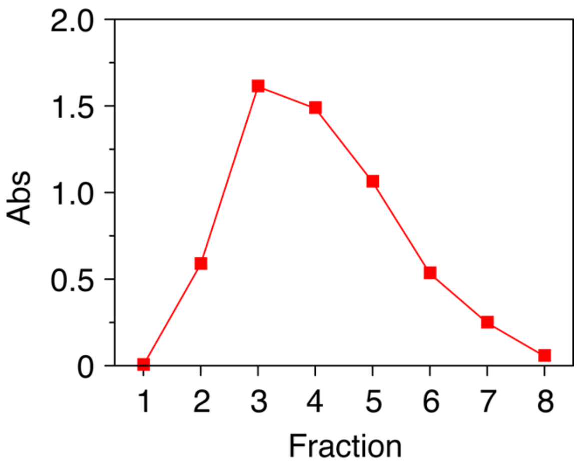

Fig. S6 SEC trace of Glue$_n$-Ph monitored at 280 nm. The sample was fractionated into 8 fractions (500 µL each), where fraction 5 was collected and identified by ^{1}H NMR as Glue$_{10}$-Ph (n in Glue$_n$-Ph represents an average number of the repeating units) (Fig. S7).

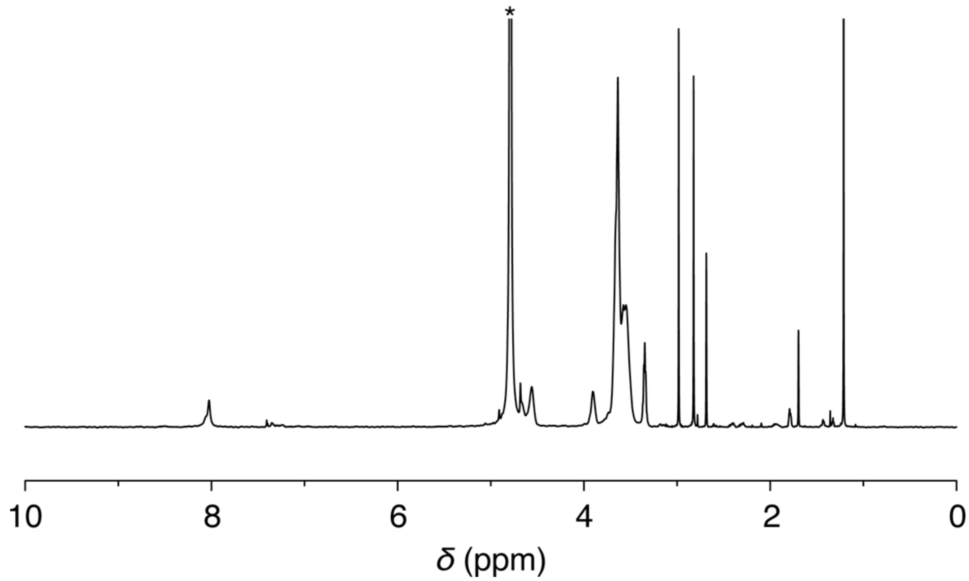

Fig. S7 ^{1}H NMR spectrum (500 MHz) of a D_2O solution of Glue$_{10}$-Ph (Fig. S6, fraction 5) at 21 °C. An asterisked signal at δ 4.79 ppm is due to water. The average number of the repeating units was estimated as 10 from the intensity ratio of signals at 2.24–2.36 ppm (2H; $CH_2CH_2CONHCH_2$) to 7.94–8.15 ppm (triazole-H).

3. Static Light Scattering (SLS) Analysis

The weight-average molecular weights (M_w) of Glue$_n$-R and ^{m}Glue$_n$-R were calculated based on the Rayleigh equation by plotting the static light scattering intensities of their aqueous solutions at various concentrations (Debye plot, Fig. S8). The plots were then fitted to the Rayleigh equation: $KC/R_\theta = (1/M_w) + 2A_2C$, where K is optical constant, C is concentration of molecular glue, R_θ is Rayleigh ratio, and A_2 is the second virial coefficient.[S1]

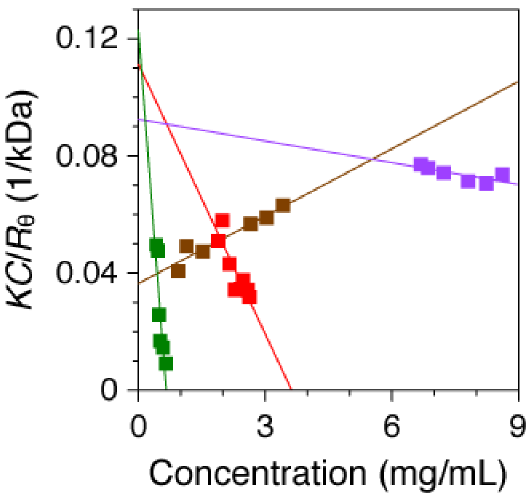

Fig. S8 Debye plots of aqueous solutions of Glue$_{29}$-BA (brown; 1.0–3.4 mg/mL), Glue$_{10}$-BA (red; 1.9–2.6 mg/mL), ^{m}Glue$_{27}$-BA (purple; 6.7–8.6 mg/mL) and Glue$_{10}$-Ph (green; 0.42–0.64 mg/mL) at 25 °C.

Table S1 Number-average molecular weights (M_n; estimated by ^{1}H NMR spectroscopy) and weight-average molecular weights (M_w; estimated by SLS analysis) of Glue$_{29}$-BA, Glue$_{10}$-BA, ^{m}Glue$_{27}$-BA and Glue$_{10}$-Ph, and their polydispersity indexes (PDI; M_w/M_n).

	M_n (kDa)	M_w (kDa)	PDI
Glue$_{29}$-BA	18.5	27.5	1.49
Glue$_{10}$-BA	6.7	9.0	1.34
^{m}Glue$_{27}$-BA	9.4	10.8	1.15
Glue$_{10}$-Ph	6.4	8.1	1.27

4. Zeta Potential Measurements

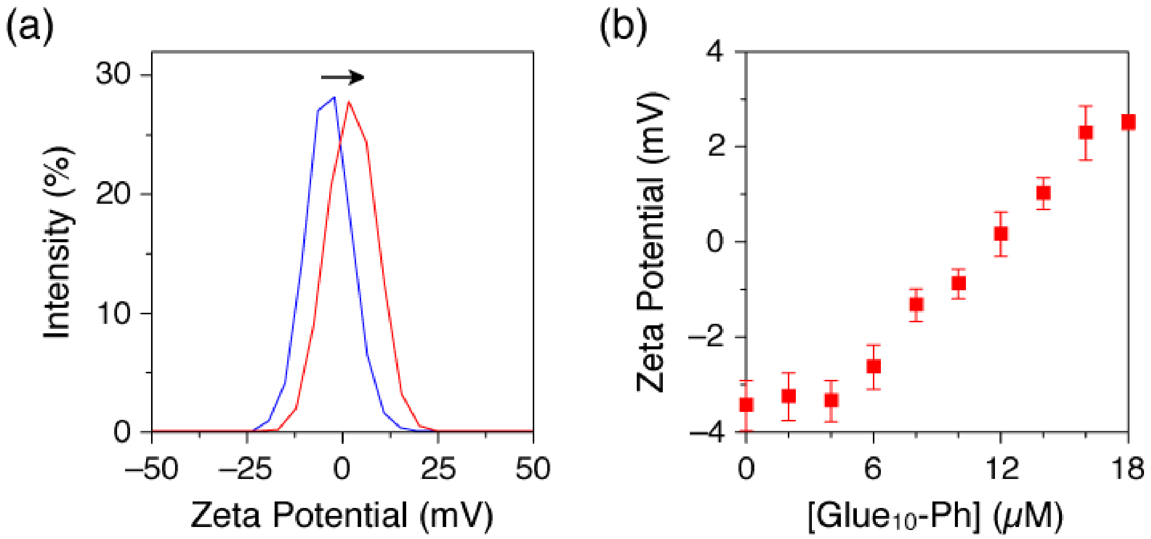

Fig. S9 (a) Zeta potential distribution profiles of trypsin (5 μM) in Tris-HCl buffer (2.5 mM Tris-HCl, 0.5 mM $CaCl_2$, pH 8.0) at 25 °C in the absence (blue solid curve) and presence of $Glue_{10}$-Ph (18 μM, red solid curve). (b) Zeta potential titration profile of trypsin (5 μM) with $Glue_{10}$-Ph (0–18 μM) at 25 °C in Tris-HCl buffer (2.5 mM Tris-HCl, 0.5 mM $CaCl_2$, pH 8.0).

=> The increase in zeta potential value from negative to positive is most likely due to charge neutralization of the carboxylate anions on trypsin by the salt-bridge formation with the positively charged guanidinium ion (Gu^+) pendants of $Glue_{10}$-Ph.

5. Circular Dichroism (CD) Spectral Titration

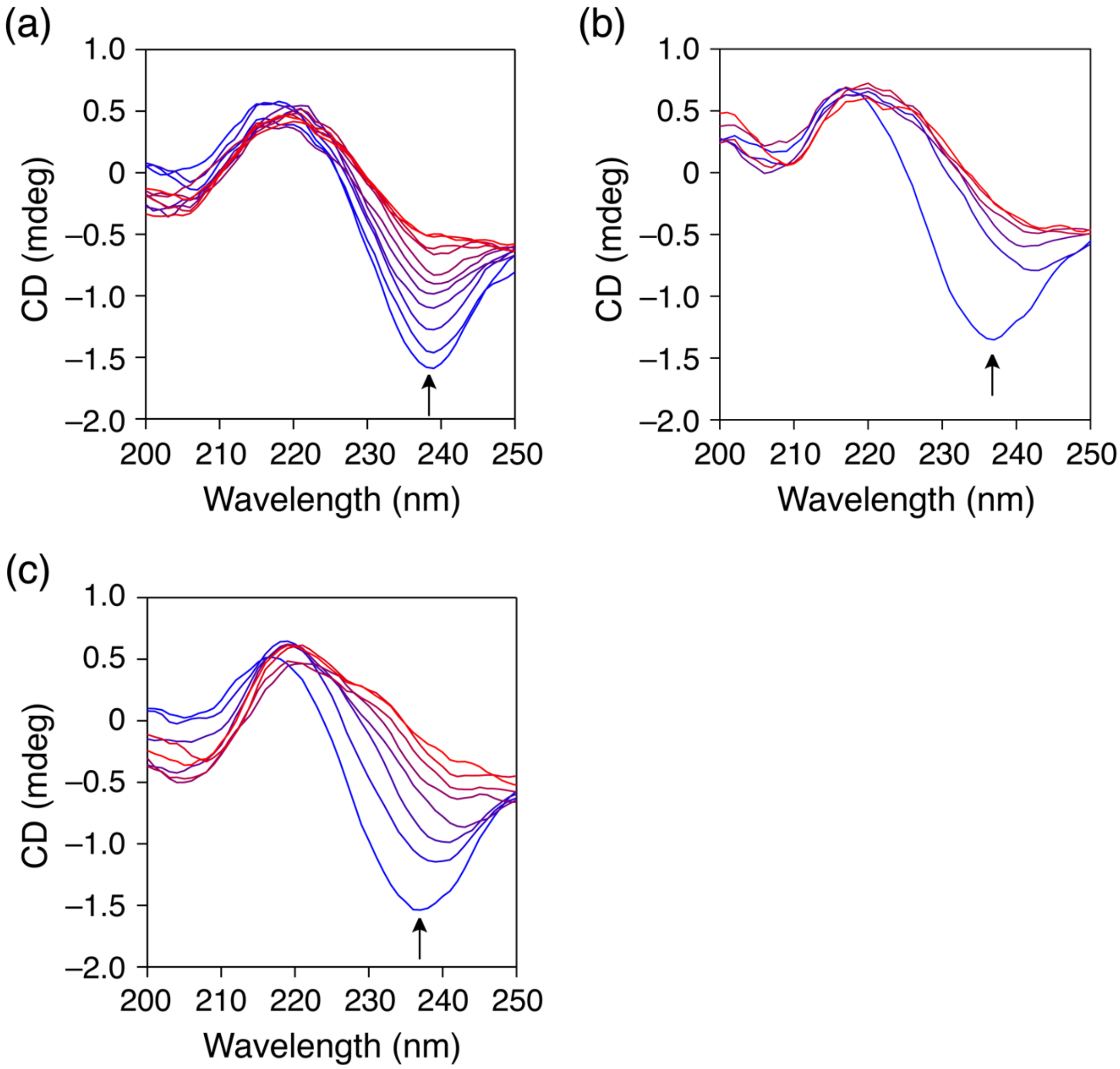

Fig. S10 CD spectral changes of trypsin (5 μM) at 25 °C in Tris-HCl buffer (50 mM Tris-HCl, 10 mM $CaCl_2$, pH 8.0) upon titration with (a) TEG-BA (0–200 μM), (b) $Glue_{29}$-BA (0–2.5 μM), and (c) $Glue_{10}$-BA (0–7 μM).

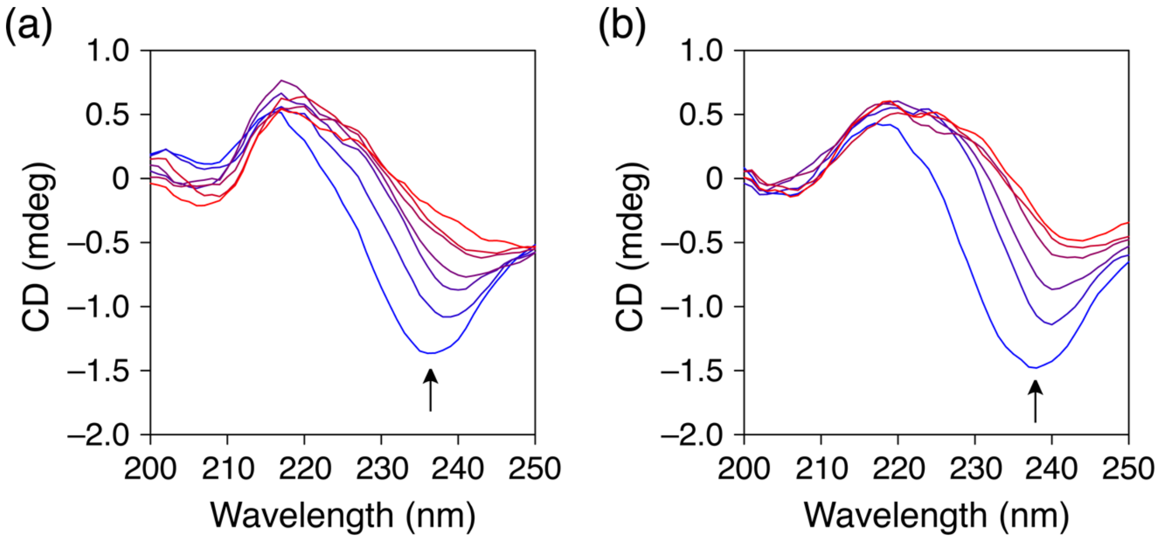

Fig. S11 CD spectral changes of trypsin (5 μM) at 25 °C in Tris-HCl buffer (50 mM Tris-HCl, 10 mM $CaCl_2$, pH 8.0) upon titration with (a) $Glue_{10}$-Ph (0–12 μM) and (b) $^{m}Glue_{27}$-BA (0–50 μM).

The association constants (K_{assoc}) were calculated based on the Hill equation by plotting the fractions of bound trypsin versus the concentrations of a titrant. The fractions of bound trypsin (θ) were calculated from $(I - I_0)/(I_{sat} - I_0)$, where I_0, I, and I_{sat} represent CD intensities at 237 nm before titration, observed with titrants, and at the saturation point, respectively. The plot was then fitted by the Hill equation: $\theta = [T]^n/((K_d)^n + [T]^n)$, where [T] is titrant concentration, K_d (= $1/K_{assoc}$) is dissociation constant, and n is Hill coefficient.[S2,S3]

6. Dynamic Light Scattering (DLS) Analysis

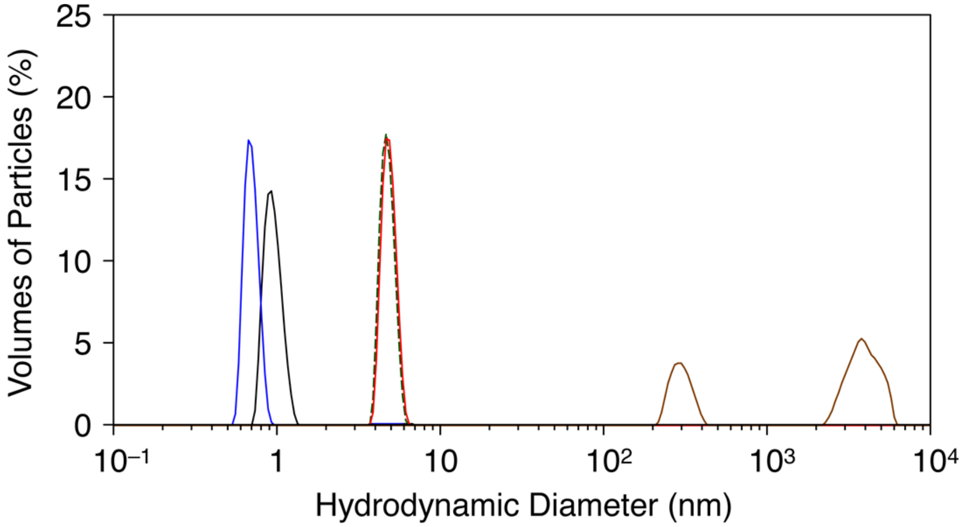

Fig. S12 DLS histograms of trypsin (5 μM) in Tris-HCl buffer (50 mM Tris-HCl, 10 mM $CaCl_2$, pH 8.0) at 25 °C before (black solid curve) and after the treatment with TEG-BA (200 μM, blue solid curve), $Glue_{10}$-BA (7 μM, red solid curve), $Glue_{10}$-Ph (12 μM, green broken curve), and $Glue_{29}$-BA (2.5 μM, brown solid curve).

7. References

S1. P. Debye, *J. Phys. Chem.*, 1947, **51**, 18.

S2. D. Hebenstreit and F. Ferreira, *Allergy*, 2005, **60**, 1208.

S3. N. J. Greenfield, *Nat. Protoc.*, 2007, **1**, 2733.
